# Hybrid Multimodal Fusion of Raw ECG Signals and Derived Measurements for Stroke Classification

**DOI:** 10.3390/bioengineering13101167

**Published:** 2026-10-07

**Authors:** Soyun Im, Chulho Kim, Yu-Seop Kim

**Affiliations:** 1 The Leading Research Center for Cerebrovascular Diseases, Hallym University, Chuncheon-si 24252, Republic of Korea; soyun012399@gmail.com; 2 Department of Neurology, Chuncheon Sacred Heart Hospital, Chuncheon-si 24253, Republic of Korea; gumdol52@hallym.or.kr; 3 Division of Software, Hallym University, Chuncheon-si 24252, Republic of Korea

**Keywords:** stroke classification, electrocardiography (ECG), multimodal learning, hybrid fusion, cross-modal interaction, deep learning

## Abstract

Stroke is a leading cause of death and long-term disability, and auxiliary methods for rapid patient identification are needed. Previous studies based on electrocardiography (ECG) have attempted stroke classification using quantitative measurements and future stroke risk prediction using raw signals. However, measurements do not preserve all waveform detail, and models using raw signals alone may not sufficiently learn quantitative characteristics from limited data. We therefore propose a hybrid fusion framework that integrates the raw 12-lead signal and the quantitative measurements of the same ECG record. It combines a signal-only model, a feature-only model, and an interaction early fusion model that couples the two inputs at the raw data and representation levels, averaging their logits for the final prediction. Evaluation used 4147 ECG records from 4020 patients in a single-institution retrospective cohort, with patient-level splits. The proposed model achieved an F1-score of 77.6%, an area under the receiver operating characteristic curve (AUC) of 0.832, and a Brier score of 0.169. Among the evaluated models, the proposed framework achieved the highest AUC and lowest Brier score. The AUC improved by 0.034 over the best unimodal model, and an AUC of 0.820 was maintained under a year-based temporal holdout. These results suggest that integrating complementary representations of the same ECG at multiple levels can improve stroke classification. The framework could potentially serve as an auxiliary decision-support tool for identifying patients who require further evaluation, using ECG characteristics statistically associated with stroke.

## 1. Introduction

### 1.1. Background

Stroke is a neurological disorder that occurs when blood flow to brain tissue is interrupted by the occlusion or rupture of a cerebral blood vessel. Ischemic stroke results from arterial occlusion caused by a thrombus or an embolus, whereas hemorrhagic stroke results from intracranial hemorrhage following vessel rupture. The consequent interruption of oxygen and nutrient supply can lead to neuronal injury and death, which may subsequently result in long-term complications such as cognitive decline, dementia, and depression [1]. Stroke is the second leading cause of death worldwide and, in terms of disability-adjusted life years (DALYs), an index that jointly reflects the burden of premature death and disability, the third leading cause of disease burden [1,2]. Between 1990 and 2021, the global number of incident strokes increased by 70.0%, stroke-related deaths by 44.0%, prevalent cases by 86.0%, and DALYs by 32.0%. The global economic burden of stroke has been estimated at approximately USD 890 billion, corresponding to about 0.66% of global gross domestic product [2]. Research aimed at the rapid recognition of stroke and timely intervention is therefore essential for reducing stroke-related mortality, disability, and economic burden.

The diagnosis of patients with suspected stroke relies primarily on neurological assessment and brain imaging such as computed tomography (CT) and magnetic resonance imaging (MRI). Blood tests and electrocardiography (ECG) are used as ancillary examinations to assess the overall condition of the patient and the potential causes of stroke [3,4,5]. ECG is a medical tool that measures the electrical activity of the heart and is widely used to detect and evaluate a range of abnormalities, including arrhythmias, pericardial and myocardial disease, electrolyte imbalance, and pulmonary disease [6]. Because stroke can cause rapidly progressing and irreversible neuronal injury, immediate recognition and treatment are critical [7]. In this respect, ECG offers the practical advantages of being simple, non-invasive, rapid, and cost-effective [6]. Previous studies have reported that ECG abnormalities, including T-wave changes, QTc prolongation, and arrhythmias, are frequently observed in patients with stroke, which suggests that ECG can serve as an ancillary tool for capturing stroke-related physiological changes [8,9,10].

### 1.2. Related Work

Several studies have attempted stroke prediction or classification using ECG. Rathakrishnan et al. [3] analyzed the statistical significance of ECG measurements in a study population comprising stroke patients with a mean age in their seventies and age-matched controls, and evaluated the performance of various machine learning classifiers. Among these, a *k*-nearest neighbors (KNN) classifier [11] achieved the highest accuracy, at 96.6%. However, because that study used an age-matched case-control sample rather than consecutively presenting clinical patients, the reported performance is unlikely to transfer directly to real clinical settings [12]. Lin et al. [13] developed an ECG-MACE model using a large-scale ECG dataset collected from clinical patients aged 30 years or older. The model adopted a ResNet-18-based multitask architecture and achieved an area under the receiver operating characteristic curve (AUC) of 0.90 for heart failure and 0.89 for all-cause mortality, whereas its performance for stroke was relatively low, with an AUC of 0.76. That task, however, concerns the prediction of future stroke occurrence and therefore cannot be compared directly with the classification task addressed in this study.

Multimodal learning has been adopted as an approach to compensate for the limitations of unimodal models. Multimodal learning jointly exploits information obtained from different forms of data to learn complementary information that is difficult to capture with a single modality [14,15]. In the medical domain, it enables richer representations for disease classification and prognosis prediction by combining heterogeneous information such as medical images, biosignals, clinical variables, and laboratory results [16]. In the ECG domain, Qiu et al. [17] integrated ECG signals with clinical information including age, sex, and comorbidities using a Transformer-based architecture to predict recurrence after atrial fibrillation ablation, achieving a significantly higher AUC than unimodal models. Similarly, Rawshani et al. [18] reported improved atrial fibrillation detection by combining ECG signals with heart rate variability (HRV) and demographic information in a convolutional neural network (CNN)-based model. Ji et al. [19] combined median-beat waveforms extracted from the ECG with 417 ECG measurements to detect acute coronary syndrome, and compared decision-level and feature-level fusion, each implemented as a separate model. More recently, hybrid fusion strategies have been proposed to jointly exploit the advantages of fusion performed at the data, representation, and prediction levels [20,21,22,23]. Previous studies have integrated multiple fusion levels within a single framework; for example, the data or representations of selected modalities were first integrated, and the resulting representation was then combined with the representations or predictions of other modalities.

### 1.3. Research Gaps and Contributions

Taken together, the studies reviewed above suggest three research gaps that warrant further investigation.

1. Relatively few studies have addressed ECG-based stroke classification in real clinical cohorts. Studies reporting high accuracy were conducted with age-matched case-control designs, a setting that differs from real clinical practice, in which patients present consecutively [12]. In addition, medical multimodal studies have mainly combined information collected from different sources, such as ECG signals, medical images, clinical text, and electronic medical records [17,24]. Because the availability and completeness of such ancillary clinical information can vary across institutions and data collection environments, approaches that rely on the ECG alone may offer complementary practical advantages [25,26].
2. Previous ECG studies on stroke have generally used one of the two forms of ECG data, either the measurements or the raw signal, as model input, in which case the information contained in the other form is not jointly exploited [27]. One study combined the waveform and the measurements of the same ECG record and compared multiple fusion levels, each implemented as a separate model [19]. Hybrid fusion strategies that combine multiple fusion levels within a single framework have also been proposed; these studies combined ECG with information from different sources, such as clinical variables and clinical notes, or addressed other types of medical data [20,21,22,23]. To the best of our knowledge, no study has yet exploited raw data-level and representation-level fusion, together with early and late fusion, within a single framework for the raw waveform and the quantitative measurements derived from the same ECG record.
3. For clinical adoption, reliable predicted probabilities and interpretable model reasoning are important in addition to discriminative performance. These aspects, however, have been examined less frequently in ECG-based multimodal studies.

To address these gaps, we propose an ECG-centric hybrid multimodal classification framework that integrates raw 12-lead ECG signals and quantitative measurements derived from the same ECG record. The classification task in this study is to distinguish stroke patients from non-stroke patients using the ECG recorded at admission; it is not to predict the future occurrence of stroke. To minimize dependence on ancillary clinical information, only sex was included among the available clinical variables. The proposed framework comprises the signal-only model, the feature-only model, and the interaction early fusion model. The interaction early fusion model learns representation-level information through a signal encoder and a feature encoder, while the raw interaction branch directly models the interaction between the raw ECG signal and the derived measurements prior to representation learning. The two modalities are therefore integrated at both the raw data level and the representation level. In the subsequent hybrid fusion stage, the predictions of the three models are combined by logit averaging to generate the final prediction. This approach is distinguished from existing methods in that it models the interaction between two complementary forms of information derived from the same ECG record at both the raw data level and the representation level, while additionally integrating, at the prediction level, the discriminative information learned independently by each model.

The main contributions of this study are as follows, each addressing the corresponding gap identified above.

1. We developed an ECG-centric hybrid multimodal framework for stroke classification using data collected from a real clinical cohort. The framework uses the raw signal and the derived measurements obtained from the same ECG record as complementary inputs and requires no ancillary clinical information other than sex.
2. We designed an interaction early fusion architecture that directly models, through a raw interaction branch, the dependency between the raw waveform and the quantitative measurements of the same ECG record at the raw data level, and combined it with the signal-only and feature-only models by logit averaging, thereby developing a hybrid fusion strategy that jointly exploits raw data-level interaction, representation-level early fusion, and prediction-level late fusion within a single framework.
3. We conducted a comprehensive evaluation, comprising comparison with pretrained ECG foundation models, statistical significance testing, calibration analysis of the predicted probabilities, year-based temporal holdout evaluation, and interpretation analyses based on Gradient-weighted Class Activation Mapping (Grad-CAM) and SHapley Additive exPlanations (SHAP), to validate not only discriminative performance but also the reliability and clinical interpretability of the predictions.

In our experiments, the proposed framework achieved the highest AUC and the lowest Brier score among the evaluated models, suggesting that integrating the complementary morphological and quantitative information of the ECG at the raw data, representation, and prediction levels can improve stroke classification.

The remainder of this paper is organized as follows. Section 2 describes the dataset, the feature sets, and their statistical characteristics. Section 3 presents the proposed framework and the evaluation metrics. Section 4 reports the experimental results, including feature set selection, model comparison, interpretation, and error analysis. Section 5 presents ablation studies, and Section 6 presents the statistical comparison, subgroup analysis, and temporal robustness analysis. Section 7 discusses the results and limitations, and Section 8 concludes the paper.

## 2. Materials

### 2.1. Dataset

**Figure 1:**
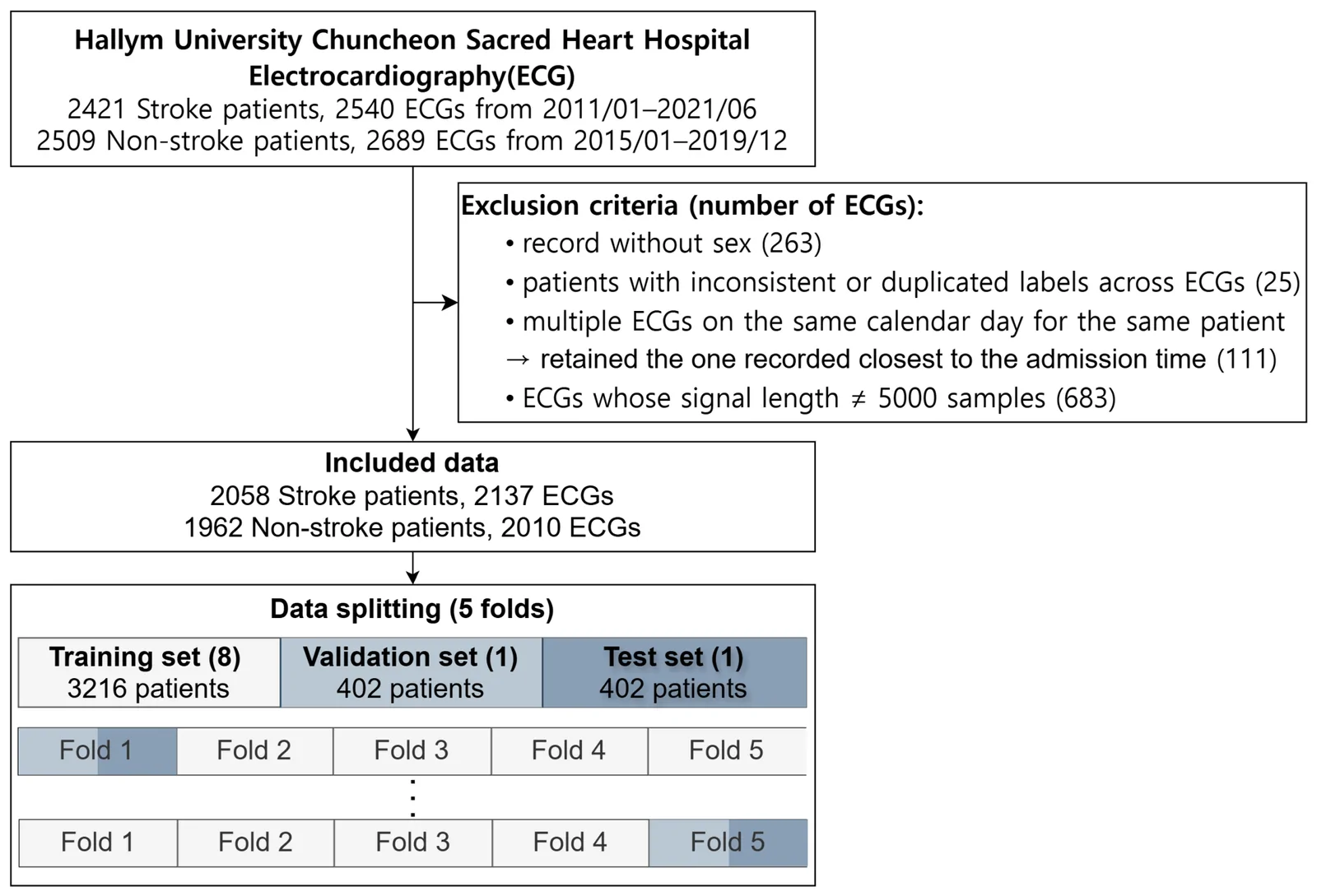
Construction process of the ECG dataset. In the data splitting diagram, white, light blue, and dark blue indicate the training, validation, and test sets, respectively.

This study used a retrospective cohort of 12-lead ECG records provided by Chuncheon Sacred Heart Hospital, Hallym University. The overall dataset construction process is summarized in Figure 1. ECGs of the stroke group were collected from January 2011 to June 2021, and those of the non-stroke group from January 2015 to December 2019. The stroke and non-stroke labels were those assigned in the clinical database of the institution that provided the data, and ischemic and hemorrhagic strokes were treated as a single class without distinguishing between subtypes. This is consistent with the intended use context of this study, in which the ECG is generally acquired before brain imaging and the stroke subtype has not yet been confirmed. The non-stroke group was selected from patients admitted to the same institution for reasons other than stroke, with no restriction on the admitting department or the admission diagnosis. Because this control group was drawn from unselected inpatients rather than from a cohort matched on age or other characteristics, it is closer to a routine hospital population than a matched case-control sample would be, although it does not specifically represent patients presenting with stroke-like symptoms. In addition, as the collection periods of the two groups were not identical, a sensitivity analysis of potential temporal confounding is presented in Section 6.3.

All ECGs were processed through the MUSE ECG Management System (GE Healthcare, Milwaukee, WI, USA). Each record contained basic clinical information, a 10 s 12-lead ECG signal sampled at 500 Hz, and ECG-derived quantitative measurements generated by the system. Among the available clinical variables, only sex was used. The experimental dataset was constructed by applying the following exclusion criteria. Records with a missing sex value, and all records of patients with conflicting stroke and non-stroke labels, were excluded. When duplicate ECGs had been recorded for the same patient on the same date, only the record acquired closest to the admission time was retained. Finally, ECG signals whose length did not match the required 5000 samples were excluded. The number of records excluded at each step is presented in Figure 1. The final dataset comprised 4147 ECG records collected from a total of 4020 patients; of these, 2137 records were obtained from 2058 stroke patients and 2010 records from 1962 non-stroke patients.

Model performance was evaluated by patient-level five-fold cross-validation. As shown in Figure 1, all patients were divided into five folds; in each iteration, one fold was split equally into a validation set and a test set, and the remaining four folds were used for training (8:1:1). All ECG records from the same patient were assigned to the same set. This study was conducted retrospectively and was approved by the Institutional Review Board of Chuncheon Sacred Heart Hospital (IRB No. 2021-07-009). Because anonymized data were used, the requirement for informed consent was waived.

### 2.2. Feature Sets

**Figure 2:**
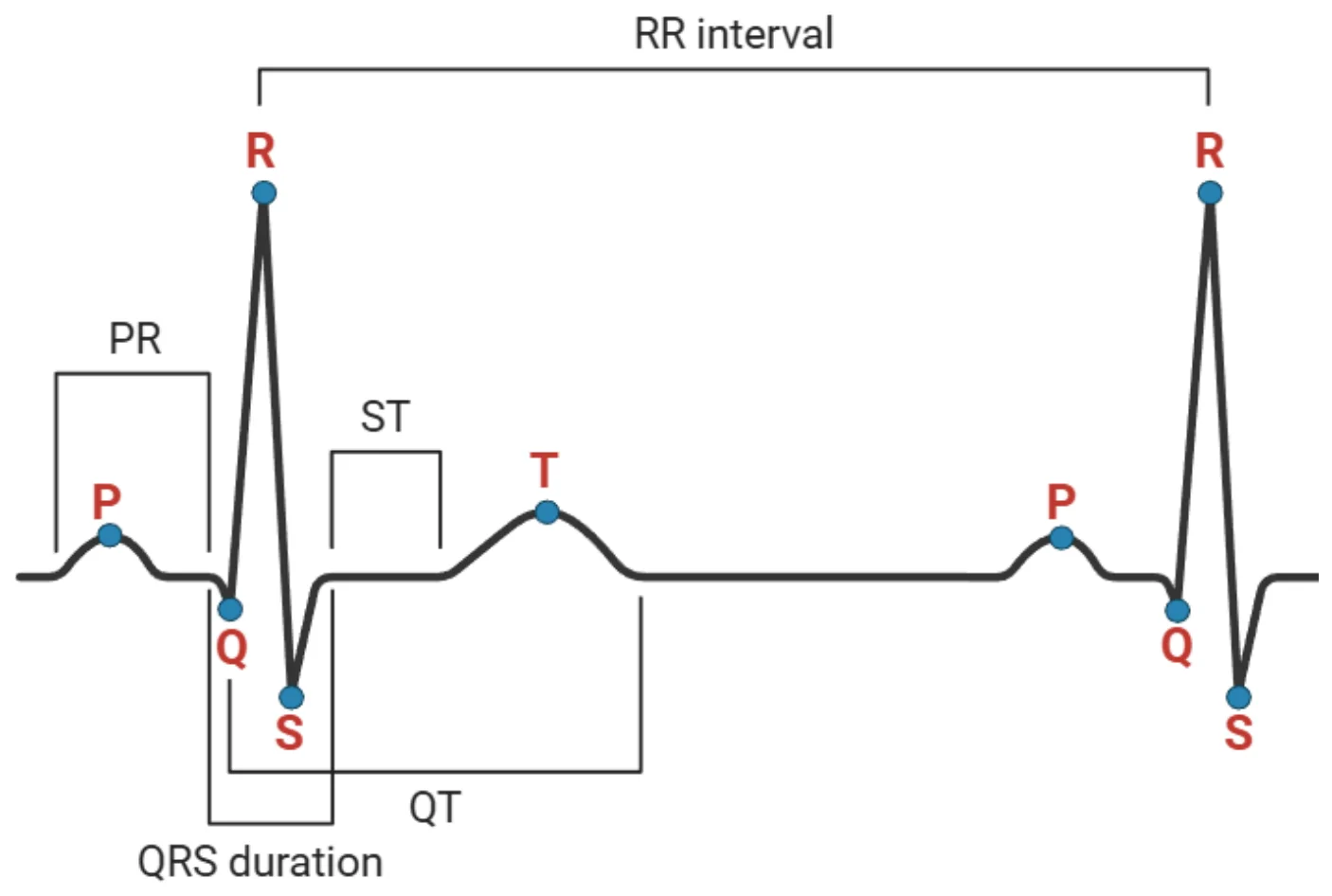
ECG signal waveform. The red letters P, Q, R, S, and T denote the principal ECG waves, and the blue dots mark the corresponding fiducial points. The brackets indicate the PR interval, ST segment, QT interval, QRS duration, and RR interval.

The ECG is a recording of the electrical activity of the heart over time and reflects the electrical and physiological state of the heart through measurements such as waveform intervals, amplitudes, and the electrical axis [28]. Figure 2 shows the principal waveform components and measurement indices of the ECG signal. The ECG waveform consists mainly of the P wave, the QRS complex, and the T wave, and the principal measurements are derived from the time intervals, segments, amplitudes, and electrical axes associated with these waveform components. Representative indices such as the PR interval, QRS duration, QT interval, ST segment, and RR interval characterize the temporal properties of cardiac electrical activity. In this study, measurements computed from the ECG records were used as the input features of the models; these ECG-derived measurements are hereafter referred to as features when they serve as model inputs. Unlike the morphological patterns learned directly from the raw signal, these features explicitly represent the time intervals and quantitative properties of cardiac electrical activity and can therefore serve as an information source that complements the signal data.

This study used both the features provided by the MUSE system and additional features extracted from the ECG signal. The names and descriptions of the two feature groups are presented in Table 1. The MUSE feature group comprises 19 quantitative features, including time intervals, durations, and electrical axes computed from the principal fiducial points of the ECG waveform. To include signal-based characteristics that the MUSE features alone represent insufficiently, 21 additional features proposed in [3] were extracted directly from the ECG signal and defined as the Extracted feature group. These features include waveform amplitudes, heart rate variability (HRV), amplitude distribution characteristics, and peak-related characteristics. Signal preprocessing and feature extraction followed the methodology presented in [3]. Noise was removed using a wavelet transform-based denoising technique, and fiducial points were detected using the Pan–Tompkins QRS detection algorithm [29].

The frequency-domain HRV features, including VLF, LF, HF, and LF/HF, were included in the initial feature set but were extracted using a separate method. Frequency-domain HRV analysis is generally performed on recordings of approximately 5 min. The ECG records used in this study, however, were ultra-short 10 s segments, which made it difficult to estimate the frequency components of RR interval variability reliably [30]. To compensate for this limitation, an autoregressive (AR) model was applied to estimate the frequency-domain HRV features. The AR method enables relatively stable spectral estimation even for short data segments [31]. Because the reliability of frequency-domain HRV features estimated from ultra-short recordings cannot be fully guaranteed, whether this feature set actually contributes to classification performance was verified through the reliability analysis and ablation experiments in Section 4.3.

In addition to the MUSE and Extracted feature groups, several further variables were constructed. Missing values in the ECG-derived quantitative measurements were imputed with medians computed on the training set only, and the same values were applied to the validation and test sets to prevent information leakage. Considering that missingness itself may carry clinically meaningful information [32], a missingness indicator (MI) variable was added for each feature containing missing values. Each MI variable was defined as 1 when the corresponding value was missing and 0 when it was observed, yielding a total of nine MI variables. In addition, because the QT interval can differ by sex [33], the clinical variable sex (denoted Gender in the MUSE system) was included in the MUSE feature group. The initial feature set therefore comprised 50 variables in total: 20 variables in the MUSE group (19 measurements and sex), 21 variables in the Extracted group, and 9 MI variables.

### 2.3. Statistical Analysis of the Dataset

**Figure 3:**
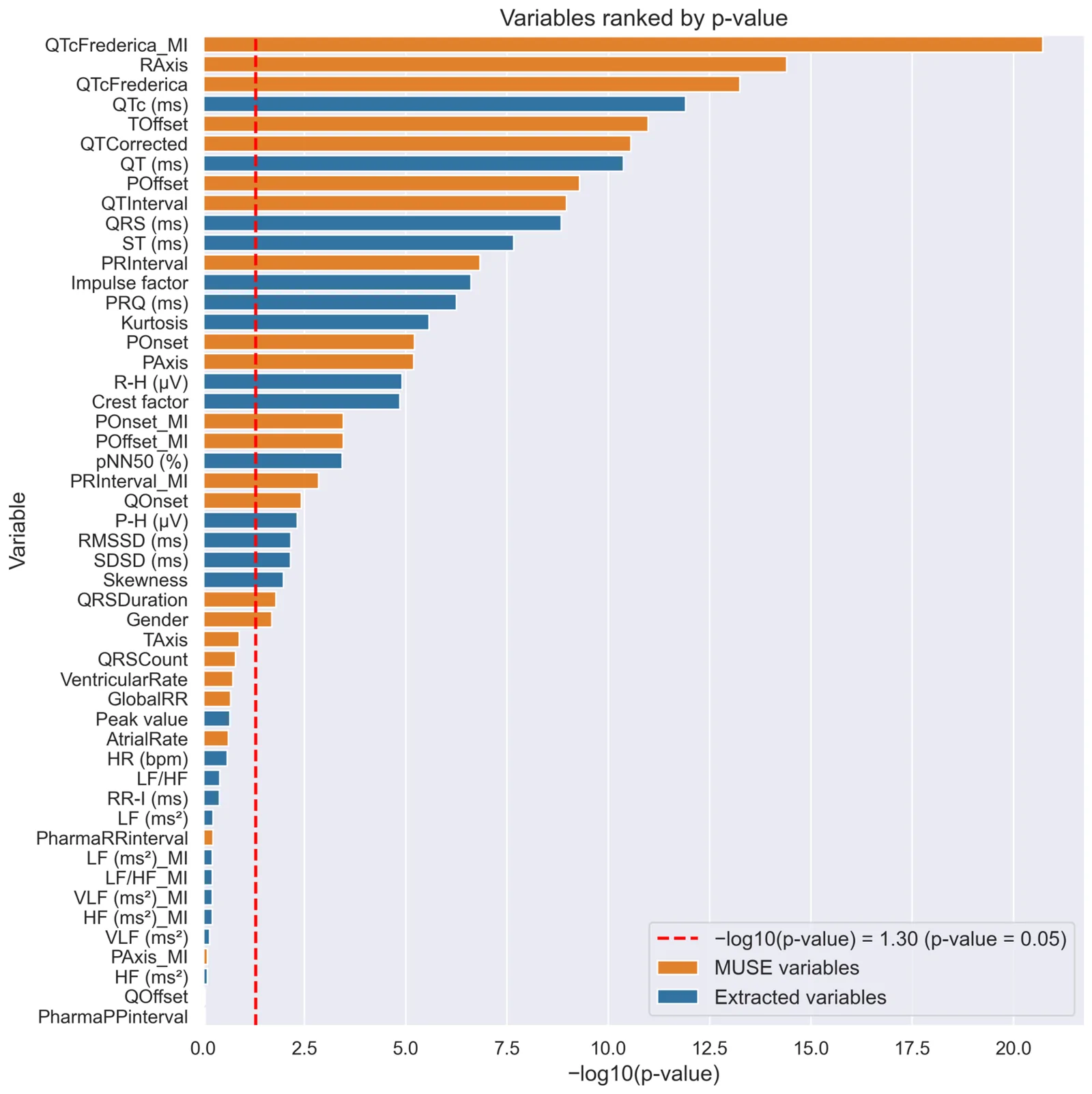
Statistical significance ranking of MUSE and extracted features.

To characterize the dataset, the 50 variables defined above were compared between the stroke and non-stroke groups. Continuous variables were evaluated using Welch’s independent-samples *t*-test [34], and categorical variables, including sex and the MI variables, using the chi-square test [35]. Table 2 presents the mean ± standard deviation of each variable in the two groups, together with the corresponding *p*-values. Figure 3 shows the ranking of the variables by statistical significance according to the *p*-value. Because the *p*-values of many features were below 0.001, the *p*-values were transformed to $-\log_{10}\left(p\text{-}\mathrm{value}\right)$ for visualization. In this transformation, smaller *p*-values are represented by larger values, so that variables with higher statistical significance appear at the top of the ranking.

Of the 50 variables, 30 differed significantly between the two groups (p<0.05), comprising 16 of the 25 variables in the MUSE group and 14 of the 25 variables in the Extracted group. The most consistent differences were observed in the QT interval-related variables: QTInterval, QTCorrected, QTcFrederica, and TOffset in the MUSE group, and QT and QTc in the Extracted group, were all longer in the stroke group. PRInterval and PRQ were also longer in the stroke group, whereas the electrical axis variables PAxis and RAxis were lower.

Among the MI variables, those related to the P wave showed higher missing rates in the stroke group, and QTcFrederica_MI showed the highest statistical significance of all variables; the implications of these missingness patterns are analyzed in Section 4.3.1. In contrast, heart rate- and RR interval-related variables, such as VentricularRate, HR, GlobalRR, and RR-I, did not differ significantly, nor did the frequency-domain HRV features (VLF, LF, HF, and LF/HF) or their MI variables. These between-group comparisons constitute an exploratory analysis intended to characterize the dataset and were not used to select the model input.

## 3. Methods

**Figure 4:**
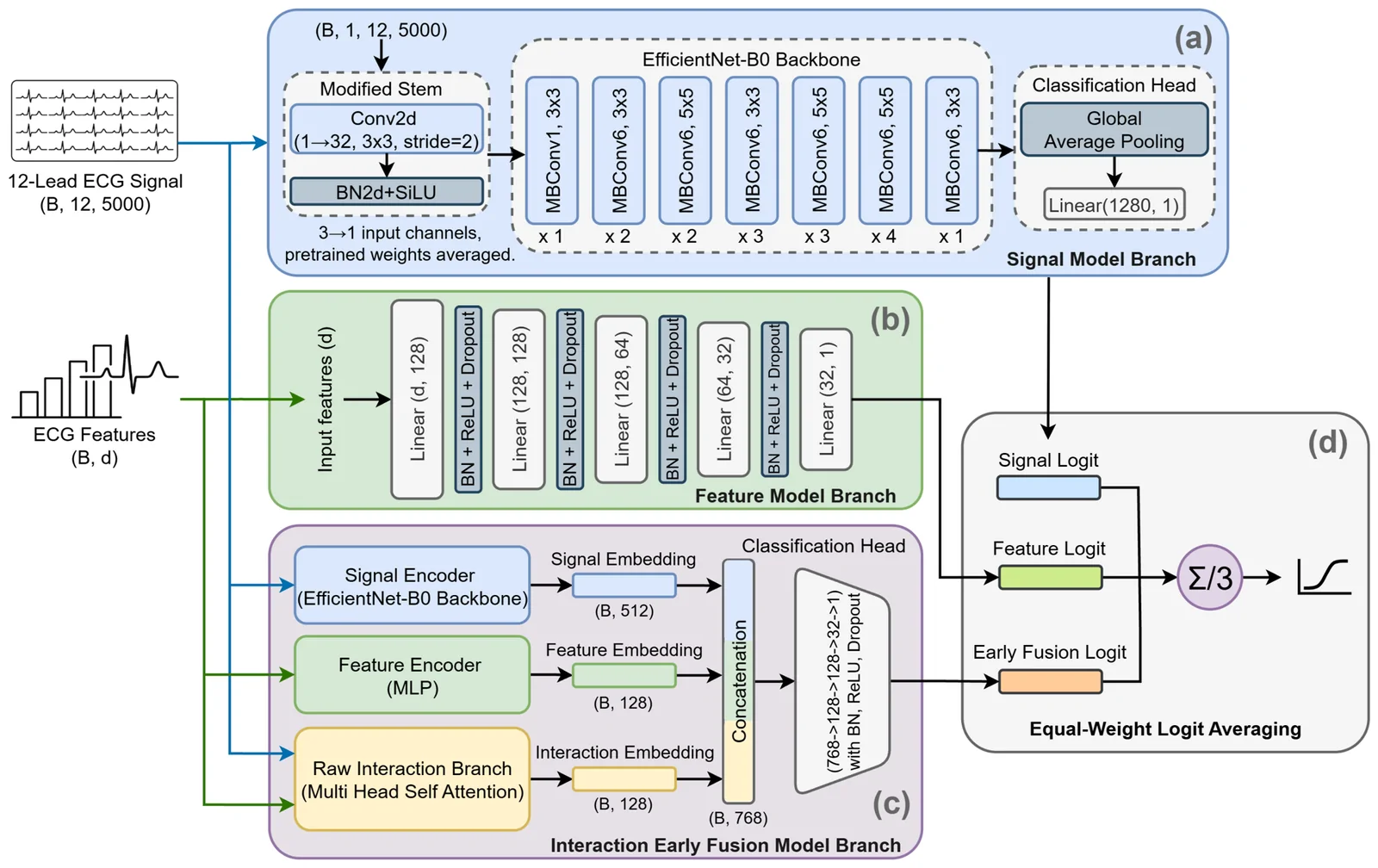
lOverall architecture of the proposed hybrid fusion multimodal ECG classification framework. (**a**) Signal classification model, used as the signal model branch. (**b**) Feature classification model, used as the feature model branch. (**c**) Interaction early fusion model, comprising the signal encoder, the feature encoder, and the raw interaction branch. (**d**) Hybrid fusion of the three branch logits for the final prediction. Blue and green arrows indicate the flow of the raw ECG signal and the ECG features, respectively, and black arrows indicate the flow of intermediate representations and logits.

In this study, an ECG-centric hybrid fusion multimodal framework was designed to effectively integrate the complementary information contained in raw 12-lead ECG signals and ECG-derived quantitative measurements. Figure 4 shows the overall architecture of the proposed framework. The framework comprises three branches. Figure 4a shows the signal model branch, which takes the raw ECG signal as input, and Figure 4b shows the feature model branch, which takes the quantitative measurements as input. Figure 4c shows the interaction early fusion model branch, which consists of a signal encoder, a feature encoder, and a raw interaction branch, through which inter-modality relationships are learned at both the raw data level and the representation level. Figure 4d shows the final prediction process, in which the final prediction is obtained by averaging, with equal weights, the signal logit, feature logit, and interaction early fusion logit generated by the three branches. The detailed architecture of each branch is described in Section 3.1, Section 3.2 and Section 3.3.

### 3.1. ECG Signal Classification Model

#### 3.1.1. Model Selection and Input Configuration

CNN-based models have been widely used for ECG signal analysis. A benchmark study using the PTB-XL dataset reported that ResNet- and Inception-based convolutional architectures achieved strong performance across several ECG classification tasks [36]. A ResNet-based CNN trained on more than two million 12-lead ECG records demonstrated that major ECG abnormalities can be classified automatically with high performance [37]. Because the ECG is inherently a time-series signal recording electrical activity over time, 1D CNN-based models provide a natural baseline for ECG classification.

A 12-lead ECG, however, is not a single time series but a set of lead signals recorded simultaneously from different directions. Accordingly, approaches that arrange the lead and time dimensions into a two-dimensional array and use it as the input to a 2D CNN have also been proposed. One study arranged the original 12-lead ECG on a two-dimensional plane and applied a ResNet-based 2D CNN to learn intra-lead and inter-lead characteristics jointly [38]. Another represented the 12-lead ECG as a two-dimensional matrix by placing each lead in a separate row and used it directly as the input to a 2D deformable CNN, without converting it into a conventional image [39].

ImageNet is a large-scale image database widely used for pretraining computer vision models [40]. A comparison of ECG classification approaches based on 1D signal inputs and 2D image representations reported that, when large-scale ECG datasets are unavailable, 2D CNNs initialized with ImageNet-pretrained weights show higher performance and greater robustness than 1D approaches [41]. More generally, vision models pretrained on images have been reported to be useful for time-series classification as well [42].

Other deep learning architectures, such as recurrent neural networks and Transformers, have also been applied to ECG analysis. Transformer-based models, however, generally require large amounts of training data to learn effective representations [43], whereas the dataset used in this study comprised approximately 4000 records. CNNs, in contrast, can effectively learn local waveform patterns such as the P wave, the QRS complex, and the T wave through their local receptive fields, and can exploit ImageNet-pretrained weights, which makes them well suited to this data condition. To examine the validity of this choice, the Transformer-based pretrained ECG foundation models ECG-FM [44] and HuBERT-ECG [45] were also included as comparison models, and the results are presented in Section 4.4.1.

Building on this previous work, candidate models for ECG signal classification were constructed using CNN-based architectures covering both 1D and 2D input representations. The models evaluated were InceptionTime [46], a 1D ResNet-18 implemented in this study based on the architecture of [13], a 2D ResNet-18 initialized with ImageNet-pretrained weights, and a 2D EfficientNet-B0 initialized with ImageNet-pretrained weights. InceptionTime is a time-series CNN model that applies multiple 1D convolutional kernels of different sizes in parallel to learn waveform patterns at various temporal scales. The 1D ResNet-18 applies the residual blocks and skip connections of ResNet to a 1D convolutional architecture [47]. EfficientNet is a family of CNN models based on a compound scaling strategy that jointly balances network depth, width, and input resolution [48].

In the 1D models, the 12 ECG leads were used as input channels, each consisting of 5000 time steps, giving an input shape of 12×5000. In the 2D models, following the representation commonly used in previous studies, the ECG signal was not converted into a conventional image and the lead–time structure of the 12-lead ECG was preserved [38,39]. Specifically, a channel dimension was added to the raw ECG array of 12 leads and 5000 time steps, giving an input shape of 1×12×5000. This representation is not intended to treat the ECG itself as an image, but to allow the model to learn temporal waveform characteristics and inter-lead patterns jointly.

To apply ImageNet-pretrained models to the ECG array, the first input layer, originally designed for three-channel RGB images, had to be adapted to the single-channel ECG input. ImageNet-pretrained vision models have been applied to single-channel spectrogram inputs by averaging the pretrained weights of the three RGB input channels [49]. Following this approach, the first convolutional layer of each 2D model was changed from three input channels to one, and its weights were initialized by averaging the pretrained weights of the original RGB channels. Because the task addressed in this study is binary classification, the final output layer of every model was configured to produce a single logit.

#### 3.1.2. Architecture of the ECG Signal Classification Model

Based on the results of the signal-based classification experiments, EfficientNet-B0 was selected as the ECG signal classification model; the comparison among the candidate models is presented in Section 4.2.1. Figure 4a shows the detailed architecture of the signal classification model. The ECG signal, converted to the required input shape, first passes through a modified stem consisting of a convolutional layer adapted to a single input channel, batch normalization, and a SiLU activation function. The resulting feature maps then pass through EfficientNet-B0, which progressively extracts the characteristics of the ECG waveform. EfficientNet-B0 uses multiple Mobile Inverted Bottleneck Convolution (MBConv) blocks as its main components. MBConv is a lightweight convolutional block designed to operate efficiently under limited computational resources. The block expands the input representation, extracts features using depthwise convolution, and then projects the representation back into a lower-dimensional space [50]. Through these blocks, the model progressively learns the morphological characteristics of the ECG waveform across the lead and time dimensions. Finally, in the classification head, global average pooling is applied to summarize the extracted feature maps into a single feature vector, from which a linear layer generates the signal logit. This model was used to evaluate signal-only classification performance and served as the signal model branch in the hybrid fusion framework.

### 3.2. ECG Feature Classification Model

#### 3.2.1. Feature-Set and Comparison-Model Configuration

Unlike the raw ECG signal, which consists of waveforms arranged continuously along the time axis, the ECG features are represented as a feature vector composed of quantitative measurements and derived variables obtained from each ECG record. To analyze the effect of feature selection and to select the feature classification model to be used as the feature model branch of the hybrid fusion framework, feature subsets of each feature group were evaluated using two classification models. The subsets of each feature group were constructed by sequentially selecting the top-ranked features according to a statistical significance ranking. To ensure that no information from the evaluation data entered the selection process, the between-group tests used for this ranking were computed independently within the training records of each split. Both the ranking and the resulting subset composition were therefore derived without reference to the corresponding validation or test records. The ranking presented in Figure 3, which was computed on the full cohort, is descriptive of the cohort and was not used to determine model input. Specifically, subsets consisting of the top 3, 5, 7, and 10 features, the set of all statistically significant features, and the complete feature set were evaluated. Because the number of statistically significant features differed across splits, the size of those subsets is reported as the mean across the five folds. For reference, the three-feature combination used in the previous study [3] was also evaluated.

A *k*-nearest neighbors (KNN) model and a multilayer perceptron (MLP) model were used for comparison. KNN was included as a baseline model because it achieved the highest performance among the traditional machine learning models evaluated for ECG feature-based stroke classification in the previous study [3]. The Euclidean distance was used for the KNN model, and the optimal value of *k* was selected using the validation set. The MLP is a neural network model that can learn nonlinear relationships among input features through fully connected layers [51]. Previous work has shown that a properly configured MLP can be applied effectively to structured numerical data [52].

In this study, the feature model had to operate not only as an independent classifier but also as the feature model branch of the hybrid fusion framework. A neural network-based model capable of producing both a final logit and an intermediate hidden representation was therefore required. From the perspective of representation learning, the intermediate representations of a neural network transform the original input into a representation that is more useful for classification [53]. Similarly, multimodal learning typically uses modality-specific branches or encoders to transform each modality into a latent representation before fusion [14,54]. Accordingly, the MLP was evaluated alongside KNN as a feature-based classification model.

In the KNN–MLP comparison presented in Section 4.2.2, the input dimension of the MLP was determined by the number of selected features. Because the subset size varied from 3 to 25, a lightweight architecture was used so that the model could be trained without overfitting even for small input dimensions. This lightweight MLP consisted of two hidden layers with 32 and 16 units, respectively, with a dropout rate of 0.2 applied after each hidden layer. This lightweight architecture was used only for the model comparison presented in Section 4.2.2.

#### 3.2.2. Architecture of the ECG Feature Classification Model

Based on the KNN–MLP comparison presented in Section 4.2.2, MLP-based models were selected for the subsequent ECG feature-based experiments. All subsequent model-based feature experiments used the feature classification architecture presented in Figure 4b. This model consisted of four hidden layers with 128, 128, 64, and 32 units, respectively, and a final linear layer that generates the feature logit. Batch normalization, a ReLU activation function, and dropout with a rate of 0.2 were applied after each hidden layer. The model was first evaluated using all 50 features and was also used in the feature removal experiments conducted to determine the optimal feature set configuration. The model using the finally selected feature set then served as the feature-only model for unimodal performance evaluation and as the feature model branch of the hybrid fusion framework.

### 3.3. Hybrid Fusion Multimodal Framework

#### 3.3.1. Early Fusion Model

Early fusion is an approach that learns an integrated representation by combining information from different modalities at an early or intermediate stage of the model. In this study, a baseline early fusion architecture that integrates the two modalities at the representation level was designed. The ECG signal and the ECG features were first transformed into a signal embedding and a feature embedding, respectively, and the two embeddings were then concatenated to form an integrated representation. In the signal branch, the 1280-dimensional vector obtained before the final linear layer of the signal classification model presented in Figure 4a was used. A linear transformation was applied to this vector to generate a 512-dimensional signal embedding. In the feature branch, the 128-dimensional vector output by the second hidden layer of the feature classification model presented in Figure 4b was used directly as the feature embedding without additional dimensional transformation. The 512-dimensional signal embedding and the 128-dimensional feature embedding were then concatenated to form a 640-dimensional integrated representation, which was passed to the classification head to generate the final prediction. The classification head consisted of three hidden layers that sequentially transform the 640-dimensional input into 128-, 128-, and 32-dimensional representations, followed by a final linear layer that generates a single logit. Batch normalization, a ReLU activation function, and dropout with a rate of 0.2 were applied after each hidden layer.

#### 3.3.2. Interaction Early Fusion Model

Whereas late fusion preserves the independent predictions of the individual unimodal models before combining them, early fusion aims to capture inter-modality interaction during representation learning. In the baseline early fusion model described above, however, the representations of the two modalities are extracted independently of each other before fusion, so the feature information cannot directly influence the early representation learning of the ECG signal. Intermediate fusion, which combines representations extracted from different modalities, has been widely used in medical multimodal classification, and among such approaches attention-based fusion is known to be an effective way to learn inter-modality relationships [55]. Self-attention was proposed as a method for computing representations by modeling the relationships among input elements [56]. Accordingly, to allow the ECG feature information to influence the ECG signal from the earliest stage of representation learning, an interaction early fusion model was designed by adding a self-attention-based raw interaction branch to the baseline early fusion model.

Figure 4c shows the overall architecture of the interaction early fusion model. In the raw interaction branch, the quantitative measurements are transformed into a representation compatible with the ECG signal input and are then processed together with the ECG signal. Self-attention is applied before the modality-specific embeddings are generated, so that inter-modality interaction is learned at an early stage. The interaction embedding generated by this branch is concatenated with the signal embedding and the feature embedding and used as the input to the final classification head. The interaction early fusion model therefore captures both the embedding-level integration performed in the baseline early fusion model and the early interaction between the ECG signal and the quantitative measurements.

**Figure 5:**
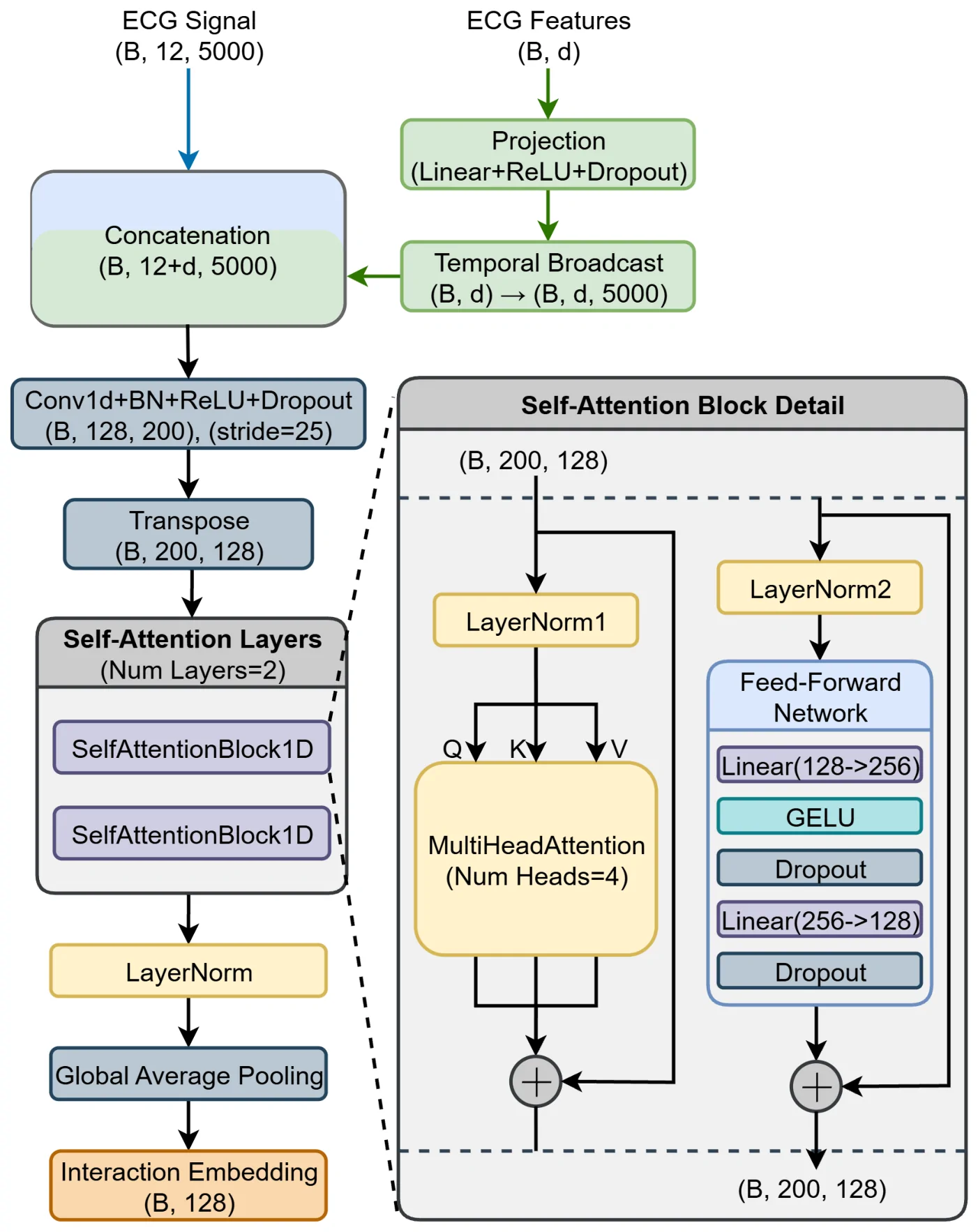
Architecture of the raw interaction branch in the proposed interaction early fusion model. Blue and green arrows indicate the flow of the raw ECG signal and the ECG features, respectively, and black arrows indicate the subsequent processing flow. The dashed lines connect the self-attention layers to an expanded view of a single self-attention block.

Figure 5 shows the detailed architecture of the raw interaction branch. The ECG signal consists of 12 leads and 5000 time steps, giving an input shape of 12×5000. The ECG feature input, in contrast, consists of *d* quantitative values and has no time dimension. The 1×d feature vector is therefore first passed through a linear projection layer to generate a *d*-dimensional learned representation. This transformation is not intended to reduce dimensionality, but to map raw measurements on different scales into a representation space in which they can be processed together with the ECG signal. The projected feature representation is then repeated identically across the 5000 time steps of the ECG signal. Through this operation, the original *d*-dimensional feature vector is transformed into a d×5000 feature matrix that has a time dimension. The transformed feature matrix is subsequently concatenated with the original ECG signal along the channel dimension. The number of channels added in this step equals *d*, the number of input features. The input shape of the raw interaction branch is therefore (12+d)×5000: the temporal length is maintained at 5000, whereas the number of channels increases from 12 to 12+d. The combined input then passes through a 1D convolutional stem layer, in which the time dimension is reduced from 5000 to 200. This downsampling reduces the computational cost of the subsequent self-attention operations. In this process, each time step is transformed into a 128-dimensional vector that jointly reflects the ECG signal and the feature information.

The resulting sequence is processed through two self-attention blocks. Each block consists of layer normalization, multi-head self-attention, and a feed-forward network. Layer normalization is first applied to the input sequence, after which multi-head self-attention learns the relationships among time steps. Because each input vector contains information from both modalities, this operation learns a combined interaction representation of the ECG signal and the quantitative measurements. The number of attention heads was set to four, allowing the model to learn different temporal relationships in parallel rather than relying on a single representational perspective. The attention output is added to the original input through a residual connection and then passes through another layer normalization layer and a feed-forward network. The feed-forward network expands the 128-dimensional representation to 256 dimensions, applies a GELU activation function and dropout with a rate of 0.2, and projects the result back to 128 dimensions. A second residual connection combines the output of the feed-forward network with its input, improving training stability. Finally, layer normalization and global average pooling over the time dimension are applied to generate a 128-dimensional interaction embedding.

The 512-dimensional signal embedding, the 128-dimensional feature embedding, and the 128-dimensional interaction embedding generated by the raw interaction branch are concatenated to form a 768-dimensional fused representation. The classification head uses the same architecture as that of the baseline early fusion model, with only the input dimension changed to 768, and generates the interaction early fusion logit. As shown in Figure 4c, this model serves as the interaction early fusion model branch of the hybrid fusion framework.

The embedding dimensions of the three branches were determined by considering both the characteristics of the input information and the results of the unimodal experiments. The ECG signal is a high-dimensional input containing the complete 12-lead waveform and provides rich temporal and morphological information about cardiac electrical activity. In the unimodal experiments, the signal-only model also achieved higher performance than the feature-only model (see Section 4.4.1). A relatively large dimension of 512 was therefore assigned to the signal embedding. The feature data, in contrast, provide explicit quantitative information derived from the ECG but yielded lower performance than the signal-only model in the unimodal experiments. In addition, the raw interaction branch does not add an independent modality but is a complementary component intended to learn the interaction between the ECG signal and the quantitative measurements. The dimensions of the feature embedding and the interaction embedding were accordingly set to 128 each. This configuration is intended to prevent representations with a relatively low independent contribution to prediction, or with a complementary role, from being disproportionately emphasized during fusion.

#### 3.3.3. Hybrid Fusion

Late fusion is a method that combines the predictions generated by different models at the final decision stage. In this study, a baseline late fusion model was constructed for comparison with the proposed model. This model generates the final prediction by averaging, with equal weights, the signal logit of the signal-only model and the feature logit of the feature-only model.

The proposed hybrid fusion model additionally integrates the interaction early fusion logit generated by the interaction early fusion model into this baseline late fusion architecture. Figure 4d shows the final prediction process of the hybrid fusion model. The hybrid fusion model comprises the signal model branch, the feature model branch, and the interaction early fusion model branch, which generate the signal logit, the feature logit, and the interaction early fusion logit, respectively. In the hybrid fusion stage, the three logits are averaged, a sigmoid function is applied to the averaged logit to obtain the predicted probability of stroke, and the final class prediction is determined using the optimal threshold selected on the validation set. Through this architecture, the final prediction reflects not only the independent predictive information learned by the unimodal models but also the interaction information between the ECG signal and the quantitative measurements learned in the interaction early fusion stage. The proposed model therefore exploits both the inter-modality interaction learning of early fusion and the prediction integration of late fusion.

The combination was performed at the logit level rather than in probability space. By combining the branch outputs before the sigmoid transformation, the differences in prediction strength that this transformation compresses were preserved. The standalone performance of the branches differed, but an appropriate combination weight depends not only on standalone performance but also on the extent to which the prediction errors of the branches overlap, and therefore cannot be determined from standalone performance alone. Estimating such weights from the data may lead to overfitting given the limited validation data available, so equal-weight averaging, which requires no additional fitting, was adopted as the default combination rule.

Logits generated by branches that use different inputs and architectures may nevertheless differ in scale and distribution, in which case a particular branch could exert a greater influence on the combined result. The distribution of each branch logit and its share of the variation in the combined logit were therefore analyzed, and equal-weight logit averaging was compared with probability averaging and with combinations based on learned weights. These results are presented in Section 5.4.

### 3.4. Evaluation Metrics

To evaluate the proposed model comprehensively, assessments were performed in terms of classification performance, the reliability of the probability predictions, and model interpretability. The primary evaluation metrics used for the overall model comparison were the AUC, the Brier score, accuracy, precision, recall, and the F1-score.

With stroke defined as the positive class, a true positive (TP) denotes a stroke record correctly classified as stroke, and a true negative (TN) a non-stroke record correctly classified as non-stroke. A false positive (FP) denotes a non-stroke record incorrectly classified as stroke, and a false negative (FN) a stroke record incorrectly classified as non-stroke. Based on these outcomes, accuracy, precision, recall (sensitivity), specificity, and the F1-score are defined as follows.

(1) $$\begin{array}{cc} \mathrm{Accuracy} & =\frac{\mathrm{TP}+\mathrm{TN}}{\mathrm{TP}+\mathrm{TN}+\mathrm{FP}+\mathrm{FN}}, \end{array}$$

(2) $$\begin{array}{cc} \mathrm{Precision} & =\frac{\mathrm{TP}}{\mathrm{TP}+\mathrm{FP}}, \end{array}$$

(3) $$\begin{array}{cc} \mathrm{Recall}\;(\mathrm{Sensitivity}) & =\frac{\mathrm{TP}}{\mathrm{TP}+\mathrm{FN}}, \end{array}$$

(4) $$\begin{array}{cc} \mathrm{Specificity} & =\frac{\mathrm{TN}}{\mathrm{TN}+\mathrm{FP}}, \end{array}$$

(5) $$\begin{array}{cc} F1\text{-}\mathrm{score} & =\frac{2\times \mathrm{Precision}\times \mathrm{Recall}}{\mathrm{Precision}+\mathrm{Recall}}. \end{array}$$

The AUC is the area under the receiver operating characteristic (ROC) curve, which plots the true positive rate (TPR) against the false positive rate (FPR) as the decision threshold varies, and is defined as follows.

(6) $$\mathrm{AUC}=\int_{0}^{1}\mathrm{TPR}\left(\mathrm{FPR}\right)\;d\left(\mathrm{FPR}\right),\;\mathrm{FPR}=\frac{\mathrm{FP}}{\mathrm{FP}+\mathrm{TN}}.$$

The AUC can be interpreted as the probability that a randomly selected positive record receives a higher predicted probability than a randomly selected negative record, and it is a threshold-independent measure of discriminative performance. The Brier score is the mean of the squared differences between the predicted probabilities and the corresponding ground-truth labels, and is defined as follows.

(7) $$\mathrm{Brier}\;\mathrm{score}=\frac{1}{N}\sum_{i=1}^{N}{\left(p_{i}-y_{i}\right)}^{2},$$

where *N* is the number of records, $p_{i}$ is the predicted probability of stroke for the *i*-th record, and $y_{i}\in \left\{0,1\right\}$ is the corresponding ground-truth label. This metric was used to assess how reliably the predicted probabilities reflect the observed outcomes; a lower value indicates closer agreement between the predicted probabilities and the actual outcomes. All evaluation results are reported as the mean across the five folds. Ratio-based metrics are expressed as percentages (%), and probability- and score-based metrics are reported as decimal values between 0 and 1.

Threshold-dependent metrics, including accuracy, precision, recall, and the F1-score, were computed on the test set using the optimal threshold determined on the validation set according to the Youden index [57]. For a given threshold *t*, the Youden index is defined as follows.

(8) J(t)=Sensitivity(t)+Specificity(t)−1.

In-depth analyses were performed to further evaluate the potential clinical applicability and the predictive interpretability of the hybrid fusion model. First, calibration curves were analyzed to assess visually the reliability of the probabilities generated by the model. A calibration curve shows the degree of agreement between the predicted risk probability and the proportion of positive outcomes actually observed. In addition, because avoiding missed high-risk cases is clinically important in stroke classification, the false positive rate at 90% sensitivity (FPR@90% sensitivity) was evaluated as an additional clinically meaningful comparison metric.

Finally, to analyze the basis on which the final proposed model generated its predictions, a qualitative interpretability analysis was performed using Grad-CAM [58] and SHAP [59]. Grad-CAM was used to visualize the regions of the ECG signal to which the model attended relatively more strongly during prediction, and SHAP was used to analyze the contribution of each input feature to the model prediction.

## 4. Results

### 4.1. Experimental Settings

The ECG foundation models ECG-FM [44] and HuBERT-ECG [45] were included as comparison models, and the basic early fusion and late fusion models implemented independently in this study were used as baselines. ECG-FM is a self-supervised model that combines contrastive and generative learning objectives, whereas HuBERT-ECG uses a masking-based self-supervised learning strategy. Both are time-series-based unimodal models that use only the ECG signal as input. Both models were initialized with the publicly available pretrained weights and then fine-tuned end to end on the dataset used in this study.

For HuBERT-ECG, a batch size of 32 and a separate learning rate of 1 × 10^−5^ were applied, as these yielded more stable training in preliminary experiments. Apart from these settings, the same training configuration was used for all models and experiments. The number of training epochs was determined using an early stopping strategy. Training was terminated when the validation loss did not improve for five consecutive epochs, and the model checkpoint with the lowest validation loss was retained. The common training settings were a learning rate of 1 × 10^−3^, a batch size of 64, and the AdamW optimization algorithm. Binary Cross-Entropy with Logits Loss (BCEWithLogitsLoss) was used as the loss function and is defined as follows.

(9) $$L_{\mathrm{BCE}}=-\frac{1}{N}\sum_{i=1}^{N}\left[y_{i}\log\left(\sigma \left(z_{i}\right)\right)+\left(1-y_{i}\right)\log\left(1-\sigma \left(z_{i}\right)\right)\right],$$

where $y_{i}$ denotes the ground-truth label, $z_{i}$ denotes the logit generated by the model, and σ(·) denotes the sigmoid function. All experiments were performed using PyTorch 2.8.0 on an NVIDIA RTX A5000 GPU, with a fixed random seed for reproducibility. All experiments used patient-level five-fold cross-validation. Performance was evaluated across the five folds, and the mean performance and the corresponding 95% confidence intervals (CIs) are reported.

### 4.2. Performance Analysis of Unimodal Models

#### 4.2.1. ECG Signal-Based Models

As shown in Table 3, the 2D models initialized with ImageNet-pretrained weights generally outperformed the 1D models in ECG signal classification. In particular, EfficientNet-B0 achieved the highest AUC (0.798) and the lowest Brier score (0.186). The 2D ResNet-18, in contrast, achieved the highest recall (79.1%) and F1-score (74.8%), showing strength in terms of sensitivity and balanced classification performance.

Both the input representation and the effect of pretraining may have contributed to these results. The 2D models in this study received the 12-lead ECG as a 12×5000 array structured along the lead and time dimensions. This representation allowed the 2D convolutional filters to learn both temporal waveform changes within individual leads and morphological patterns occurring simultaneously across different leads. Although 1D CNNs can also integrate information across input channels, 2D CNNs use kernels that move along both the lead and time dimensions and can therefore learn local relationships across the two axes more explicitly.

In addition, despite the domain gap between natural images and ECG signals, ImageNet-pretrained weights provide filters that are already optimized to detect basic patterns such as edges, transitions, and local structures. Compared with training a model from scratch on a limited ECG dataset, such initialization may have contributed to more stable optimization and improved generalization. The superior performance of the 2D models observed in this study can therefore be interpreted as the combined result of a 2D representation that preserves the lead–time structure and the stable initialization provided by ImageNet pretraining. However, because the input dimensionality, the model architecture, and the use of pretraining all differed simultaneously in this comparison, the observed performance differences cannot be attributed exclusively to either the 2D architecture or ImageNet pretraining.

The 2D ResNet-18 attained higher recall and F1-score, whereas EfficientNet-B0 was superior not only in accuracy and precision but also in the threshold-independent AUC and Brier score. Recall and the F1-score can be adjusted through the classification threshold, whereas the AUC and the Brier score reflect the underlying discriminative ability and the quality of the probability estimates independently of the threshold. Giving priority to overall discrimination and probability quality over performance at a particular threshold, EfficientNet-B0 was selected as the default signal-only model for the subsequent fusion experiments.

The difference between the two models was nevertheless small, at 0.005 in AUC and 0.001 in Brier score, so these results alone do not establish that EfficientNet-B0 is clearly superior to the 2D ResNet-18. As shown in Section 5.3, computational efficiency likewise favored the two models in opposite directions: EfficientNet-B0 has fewer parameters, whereas the 2D ResNet-18 was more efficient in training time, memory use and inference latency. EfficientNet-B0 was therefore adopted as the default signal encoder while the 2D ResNet-18 was retained as a competitive alternative. In particular, the signal encoder is instantiated in both the signal model branch and the interaction early fusion branch. Section 5.3 therefore compares the two encoders within the complete framework, in terms of both classification performance and computational complexity, to determine whether the small standalone difference is preserved or amplified.

In summary, the ImageNet-pretrained 2D models generally outperformed the 1D models in ECG signal classification. Among them, EfficientNet-B0 achieved the highest AUC (0.798) and the lowest Brier score (0.186) and was selected as the signal-only model, although its performance difference from the 2D ResNet-18 was small.

#### 4.2.2. Feature-Based Models

The performance of KNN and MLP across the feature subsets of each group is presented in Table 4 and Table 5, respectively. The MLP outperformed KNN, achieving the higher AUC in 12 of the 13 configurations evaluated. This is likely because KNN is a distance-based classifier that relies primarily on distances between records, whereas the MLP can learn complex relationships among features through nonlinear transformations. Consistent with this, the AUC gap between the two models widened with the number of input features, from a mean of 0.012 for configurations with seven or fewer features to 0.026 for those with ten or more. The MLP was therefore selected as the feature classification model.

The MUSE feature set also outperformed the Extracted feature set. Both KNN and the MLP achieved their highest AUC with the MUSE All feature set. In particular, the MLP recorded the highest AUC, 0.694, and the lowest Brier score, 0.220, among the configurations compared in Table 5. This difference may reflect the way the two feature sets are measured and derived. The MUSE features consist of fiducial point-based variables generated directly by the ECG system and include waveform intervals, durations, onsets, and offsets. Such features can represent the morphological and temporal characteristics of the ECG relatively consistently. The Extracted features, in contrast, are obtained through separate signal preprocessing and feature extraction, and may therefore be affected by the variability introduced in those steps. The three-feature combination used in the previous study [3], comprising LF/HF, ST and QT, recorded AUCs of 0.523 and 0.549 with KNN and the MLP, indicating limited discriminative performance on this dataset.

In the feature set evaluation using the MLP, the complete feature set achieved the highest AUC and the lowest Brier score within both the MUSE and the Extracted group. The number of features satisfying the p<0.05 criterion was 15.4 and 12.4 on average across splits, and the AUC of these subsets was lower than that of the complete set by 0.003 and 0.008, respectively. Both differences were smaller than the half-width of the corresponding confidence intervals (0.024–0.039). Recall, in contrast, was higher for the significance-based subset in both groups, and the F1-score was also higher in the Extracted group. The effect of significance-based feature selection therefore varied with the evaluation metric. Accordingly, the complete set of 50 features from both groups was compared with subsets constructed according to the significance ranking, and a configuration retaining all MUSE features while applying the significance criterion to the Extracted group only was additionally evaluated.

Table 6 shows how classification performance varied across the feature sets constructed from the two combined feature groups. The AUC improved as the number of input features increased, and the All feature set achieved the highest AUC of 0.720 and the lowest Brier score of 0.213. Accuracy and the F1-score were also highest for the All feature set, at 66.3% and 68.1%. The configuration applying the p<0.05 criterion to both groups recorded an AUC of 0.692, and the configuration combining all MUSE features with the significant Extracted features recorded 0.703. These results suggest that using all available features may be advantageous compared with restricting the input to statistically significant features or to features from the better-performing group only.

Precision, however, was highest for the MUSE All + Extracted p<0.05 configuration, and recall was highest for the configuration using the top 10 features. Increasing the number of features therefore did not improve every evaluation metric consistently. In addition, a univariate statistical test evaluates the between-group difference of each feature individually and does not directly reflect its contribution when used together with other features in a multivariate model. The complete feature set was accordingly used as the basis for the subsequent analyses, while the validity and reliability of the individual feature groups were examined separately through feature analysis and removal experiments.

In summary, the MLP achieved a higher AUC than KNN in 12 of the 13 configurations and was therefore selected as the feature classification model. In addition, using all 50 features, rather than restricting the input features by statistical significance, yielded the highest AUC (0.720) and the lowest Brier score (0.213).

### 4.3. Optimal Feature Set Selection Through Feature Analysis and Ablation

Before constructing the optimal feature set, the validity of the MI variables, the reliability of the HRV-related features, and the potential redundancy between the MUSE and Extracted feature groups were assessed. Ablation experiments were then performed in which each feature set was selectively excluded, so that the effect on classification performance could be compared and the final feature set to be used as the model input could be determined.

#### 4.3.1. Missingness Analysis

To assess the validity of including the MI variables, the missingness mechanism was analyzed from the perspectives of missing completely at random (MCAR), missing at random (MAR), and missing not at random (MNAR) [60]. Little’s MCAR test was performed first [61], and the MCAR hypothesis was rejected in both groups (p<0.05). This indicates that the missing values did not occur completely at random.

Next, the difference in missing rates between the two groups was compared to assess the association between the MI variables and the class label. Table 7 summarizes the differences in feature-wise missing rates between the stroke and non-stroke groups. The MI variables for QTcFrederica, POnset, POffset, and PRInterval differed significantly between the two groups, with higher missing rates observed in the stroke group. The difference in the missing rate for PAxis, in contrast, was very small at 0.003 and was not statistically significant. Similarly, the HRV-related features, including LF, HF, VLF, and LF/HF, had identical overall missing rates, and the difference between the stroke and non-stroke groups was only 0.006 and was not statistically significant.

Univariate associations between the MI variables and age, sex, and acquisition year were then analyzed. Table 8 presents the MI variables for which the data acquisition-related factors yielded an AUC of 0.60 or higher in predicting missingness. For each MI variable, the associated factor and its level, the odds ratio (OR), the *p*-value, and the AUC are reported. The OR indicates the direction and magnitude of the change in the odds of missingness; a value greater than 1 indicates an increased likelihood of missingness, and a value less than 1 a decreased likelihood.

The analysis showed that the QTcFrederica MI variable was very strongly associated with the acquisition year (AUC 0.976). Missingness in QTcFrederica was observed only in the stroke group and therefore differed significantly between classes, but this difference was more likely to arise from the different data collection periods of the two groups than from the disease state itself. This missingness pattern was therefore judged to be more appropriately interpreted as MAR.

The MI variables for POnset, POffset, PRInterval, and PAxis, in contrast, were significantly associated with age, with AUCs ranging from 0.712 to 0.715. Because some of these variables were also significantly associated with the class label, missingness in the P-wave-related features may reflect a non-random pattern related to age and disease state rather than a simple technical omission. Clinically, this may indicate that P-wave measurement became unstable because of age- or disease-related changes [62], which suggests a possible MNAR pattern.

The MI variables for the HRV-related features, on the other hand, showed almost no difference between classes and no clear or significant association with age, sex, or acquisition year. This suggests that missingness in the HRV-related features is more likely to follow a MAR pattern arising from procedural factors such as the technical limitations of the feature extraction process.

Taken together, the missingness analysis showed that the QTcFrederica MI variable was primarily related to the acquisition year. In contrast, missingness in the P-wave-related features was associated with age and disease state, whereas that in the HRV-related features was more consistent with procedural factors. The QTcFrederica MI variable was therefore excluded, and the remaining 49 features were defined as the baseline feature set. Using this baseline as the reference, ablation experiments were performed in which the P-wave-related MI variables, the HRV-related MI variables, and all MI variables were each removed, so that the effect of each feature group on classification performance could be evaluated.

#### 4.3.2. HRV Feature Reliability

The preceding missingness analysis indicated that missingness in the HRV-related features is more consistent with a MAR pattern arising from the technical limitations of the feature extraction process than with a disease-specific pattern. For the HRV-related variables, however, not only missingness but also the reliability of the estimated feature values must be considered. Frequency-domain HRV variables such as LF, HF, VLF, and LF/HF generally require a sufficiently long RR interval time series for stable estimation. Because the ECG records used in this study were ultra-short recordings of only 10 s, the stability of these estimates could not be fully guaranteed [63]. We therefore analyzed the within-record variability using overlapping 8 s windows, which retain most of the original recording while allowing the effect of a different starting point to be assessed.

As shown in Table 9, the median CV was 0.565 to 0.744 for VLF, LF and HF, approximately three to six times higher than the 0.133 to 0.191 observed for the time-domain HRV features. The median CV of the LF/HF ratio was 0.402, the lowest of the four features, but still higher than that of the time-domain features. For VLF, LF and HF, the standard deviation across windows exceeded the within-record mean of the estimates in 34.9%, 32.3% and 25.8% of records, respectively. By contrast, the median CV was 0.013 for both the mean RR interval and heart rate, and no record showed a standard deviation larger than its mean. The CV of the frequency-domain features could moreover be computed for only 3367 of the 4147 records, whereas the time-domain features were obtainable for essentially all records. These results indicate that the frequency-domain HRV features were sensitive to window position even within the same 10 s ECG and showed lower estimation stability than the time-domain features.

In summary, the frequency-domain HRV features showed approximately three to six times higher within-record variability than the time-domain features, and the standard deviation across windows exceeded the mean in roughly one quarter to one third of records, indicating low estimation stability in 10 s ECGs.

Building on these results, the effect of this estimation instability on classification performance was assessed further. To this end, an ablation experiment was performed in which the frequency-domain HRV features were removed. This feature set comprised eight features in total: LF, HF, VLF, LF/HF, and the corresponding MI variables. The detailed results are presented in Section 4.3.4.

#### 4.3.3. Multicollinearity Analysis

Finally, a correlation analysis was performed to examine the potential redundancy between the MUSE and Extracted features. Table 10 summarizes the feature pairs with an absolute correlation coefficient (|r|) of 0.80 or higher between the MUSE and Extracted feature groups, together with their physiological interpretation. The analysis identified high correlations involving two features of the Extracted group and four features of the MUSE group.

Specifically, HR in the Extracted group was strongly positively correlated with VentricularRate and QRSCount in the MUSE group, and strongly negatively correlated with GlobalRR and PharmaRRinterval. Similarly, RR-I in the Extracted group was strongly positively correlated with GlobalRR and PharmaRRinterval but strongly negatively correlated with VentricularRate and QRSCount. These results suggest that some features in the two groups redundantly represent similar information about heart rate and the RR interval.

Not all conceptually similar variables, however, were highly correlated. For example, QT in the Extracted group and QTInterval in the MUSE group represent the same QT interval, yet their absolute correlation coefficient was below 0.80. This may be because variables representing the same physiological concept were computed using different measurement methods or calculation procedures. In other words, some variables may not provide numerically identical information even when they are conceptually similar. Nevertheless, the high correlations observed for several feature pairs indicated potential redundancy.

In summary, high correlations between the MUSE and Extracted groups were observed for heart rate- and RR interval-related features, indicating potential redundancy, whereas conceptually identical variables such as the QT interval could still provide different information depending on how they were computed. Ablation experiments removing the highly correlated feature sets were therefore performed to evaluate the effect of this redundancy on classification performance.

#### 4.3.4. Feature Configuration Ablation

To determine the final input feature configuration, feature group ablation experiments were performed, focusing on the MI variables, the HRV feature set, and the redundancy between features from different sources. Table 11 shows how classification performance changed when specific feature groups were removed from the reference feature set. The baseline was defined as the refined set of 49 features excluding the QTcFrederica MI variable, and its AUC was 0.701. The MI only configuration, which used only the MI variables, performed close to chance level with an AUC of 0.514, and removing all MI variables increased the AUC to 0.709. Taken together, these two results indicate that the model did not use missingness itself as a primary discriminative cue and that including the MI variables is not always beneficial.

Removing the frequency-domain HRV features and their corresponding MI variables increased the AUC to 0.718, the highest value among all configurations. This is consistent with the low estimation stability identified in the preceding within-record variability analysis, and suggests that these features did not reliably provide additional discriminative information under the present data conditions. When the two individually beneficial strategies, removing all MI variables and removing the HRV feature set, were applied together, however, the AUC decreased to 0.705, which is lower than that obtained by removing the HRV feature set alone. This suggests that the P-wave-related MI variables, excluding the HRV-related ones, may still have provided complementary information when combined with the other features.

In the redundancy-related comparisons, removing the redundant MUSE features and removing the redundant features of both groups yielded AUCs of 0.704 and 0.702, respectively, slightly higher than the baseline. Removing the redundant Extracted features, in contrast, yielded an AUC of 0.697, lower than the baseline. This shows that removing redundant variables can help maintain or slightly improve performance, but that the result depends on which group of variables is removed. In addition, applying the two individually beneficial strategies together, removing the HRV feature set and removing the redundant MUSE features, yielded an AUC of 0.716. This value was higher than the baseline but lower than the AUC obtained by removing the HRV feature set alone. These results show that reducing redundancy can be partly useful but that removing redundant features does not always lead to further performance gains.

These results show that not all numerical variables are equally useful for classification and that some unstable or redundant variables may in fact degrade performance. Moreover, combining individually beneficial feature removal strategies did not always yield additional benefits, which suggests that optimizing the feature configuration is not simply a matter of reducing the number of variables. Rather, a balance must be sought between noise reduction and the preservation of complementary information.

Overall, the configuration excluding only the HRV feature set achieved the best performance in terms of AUC and Brier score. Accordingly, the four HRV features and the four corresponding MI variables were excluded from the 49 baseline features, yielding an input feature set of 41 features in total. The same 41-feature set was used as the input to the feature-only model and to the multimodal models in all subsequent experiments.

### 4.4. Performance Comparison and Model Interpretation

#### 4.4.1. Performance Comparison Between Baseline and Proposed Models

Table 12 compares the classification performance of the unimodal models, the pretrained ECG foundation models, the baseline multimodal models, and the proposed hybrid fusion model. Among the unimodal models, the signal-only model achieved the best performance, with an AUC of 0.798. The feature-only model, in contrast, showed relatively low discriminative performance, with an AUC of 0.718. The pretrained ECG foundation models ECG-FM and HuBERT-ECG achieved AUCs of 0.717 and 0.761, respectively, both lower than that of the ImageNet-pretrained 2D CNN-based signal-only model. These results show that, for the dataset and experimental setting of this study, the 2D CNN-based approach may have been more effective at capturing subtle morphological patterns of the ECG signal than the ECG foundation models evaluated.

The baseline multimodal models generally outperformed the unimodal models. The baseline early fusion model achieved an AUC of 0.810, and the late fusion model, which combines the predictions of the signal-only and feature-only models, achieved an AUC of 0.818. The interaction early fusion model, a constituent branch of the hybrid fusion model, achieved an AUC of 0.815, higher than that of the baseline early fusion model. These results suggest that ECG signals and quantitative measurements provide complementary information and that integrating the two representations improves classification performance relative to using either unimodal input alone.

The proposed hybrid fusion model achieved an accuracy of 75.5%, a precision of 73.5%, a recall of 82.7%, an F1-score of 77.6%, an AUC of 0.832, and a Brier score of 0.169, the best overall performance among all models evaluated. These results suggest that combining different fusion strategies, rather than applying a single fusion method, contributed to exploiting the multimodal information effectively and improving classification performance. Hybrid fusion recorded the lowest FPR at 90% sensitivity, 45.2%, compared with 47.2% for late fusion and 52.7% for the signal-only model. This means that the proposed model produced fewer false positive predictions while maintaining a high level of sensitivity.

**Figure 6:**
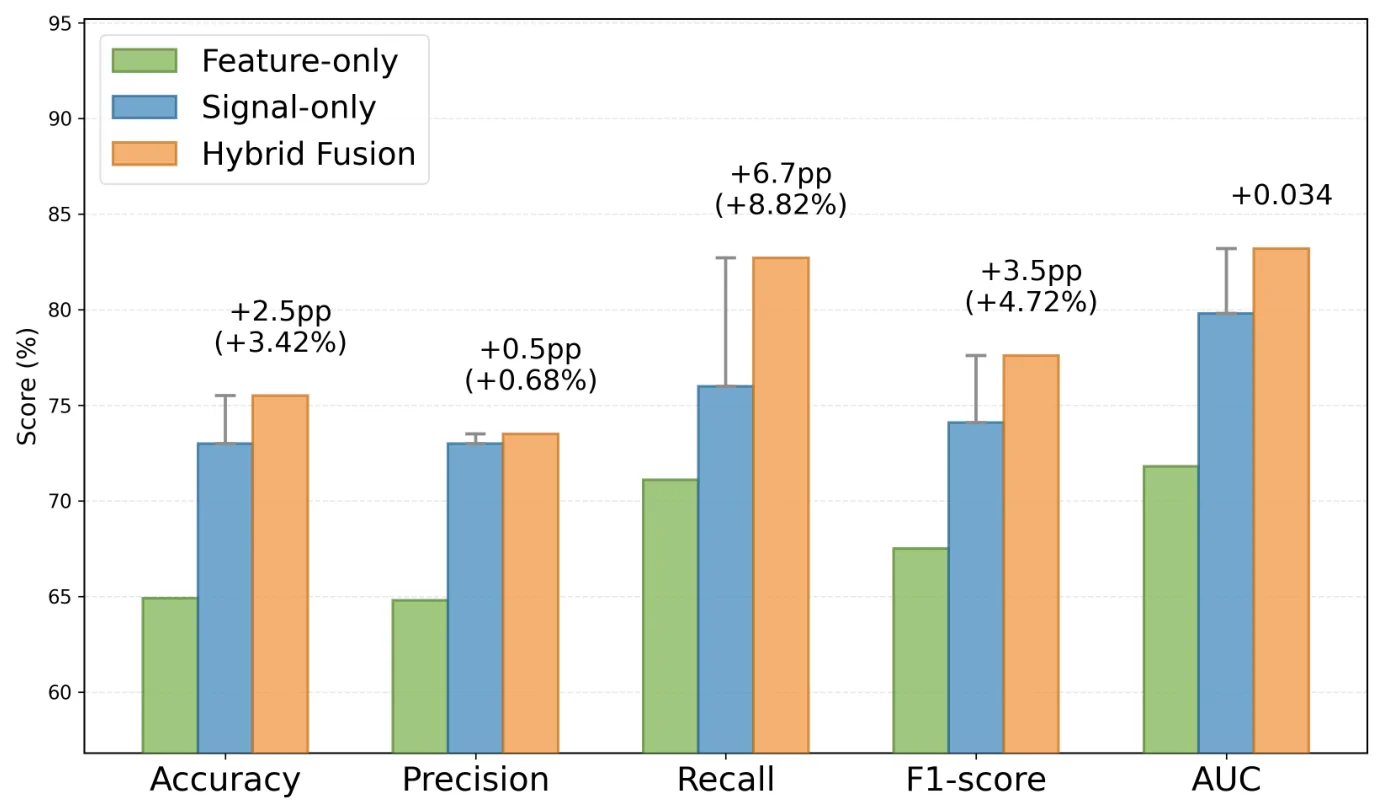
Performance comparison between unimodal and multimodal models.

Figure 6 shows the overall performance improvement of the proposed hybrid fusion model relative to the signal-only model. Compared with the unimodal signal-only model, hybrid fusion improved accuracy by 2.5 percentage points, recall by 6.7 percentage points, the F1-score by 3.5 percentage points, and the AUC by 0.034. The Brier score also decreased by 0.017, and the FPR at 90% sensitivity decreased by 7.5 percentage points. The improvement in precision, however, was limited to 0.5 percentage points, indicating that the performance gain of the proposed model derived mainly from the improvement in recall.

**Figure 7:**
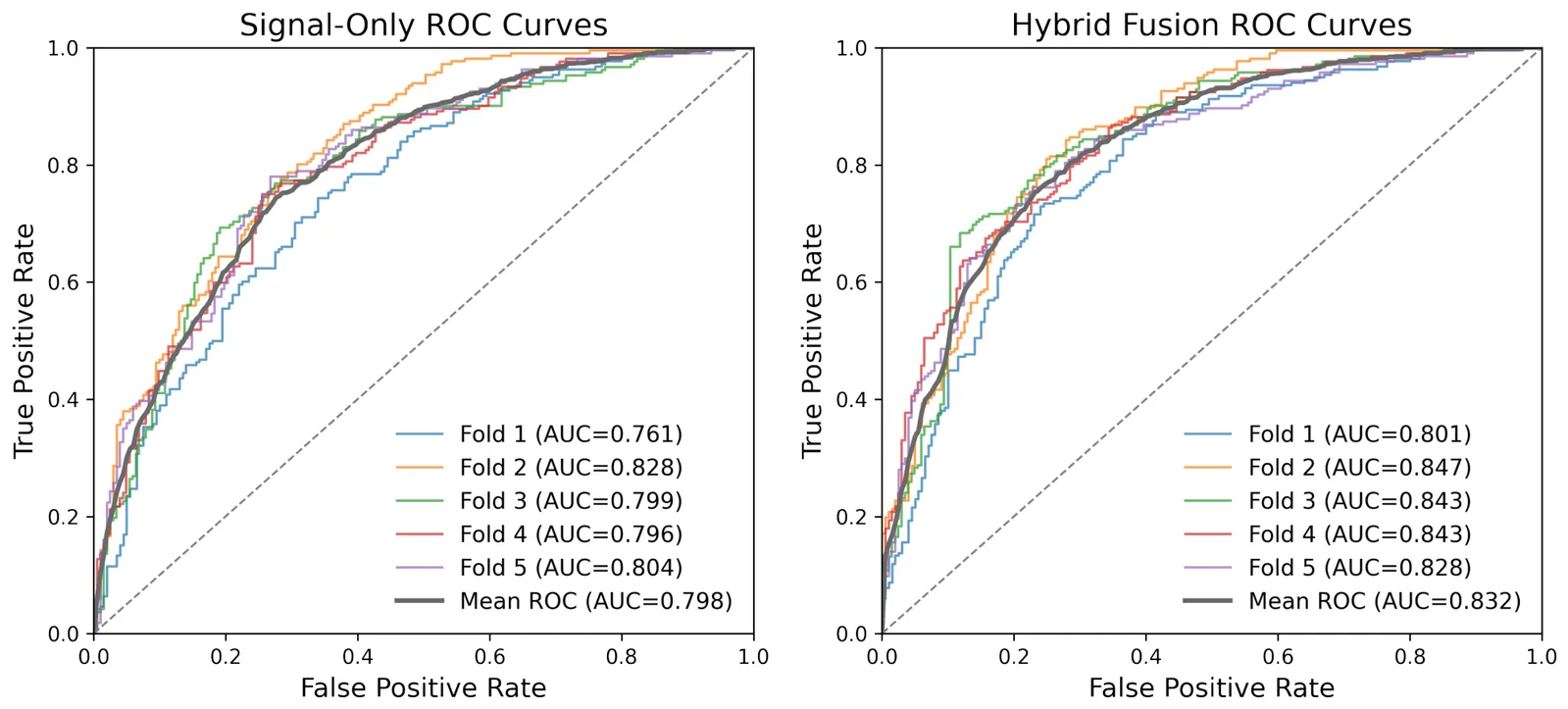
Receiver operating characteristic (ROC) curve comparison between the signal-only and hybrid fusion models.

ROC and calibration curves were analyzed to further assess the consistency of model performance and the reliability of the probability estimates. As shown in Figure 7, hybrid fusion achieved a mean AUC of 0.832 in five-fold cross-validation. The ROC curves of the individual folds were distributed relatively closely, indicating that the model maintained consistent discriminative performance across different data splits. The ROC curve of hybrid fusion was also located closer to the upper-left corner overall than that of the signal-only model, demonstrating the improved discriminative ability of the proposed multimodal architecture.

**Figure 8:**
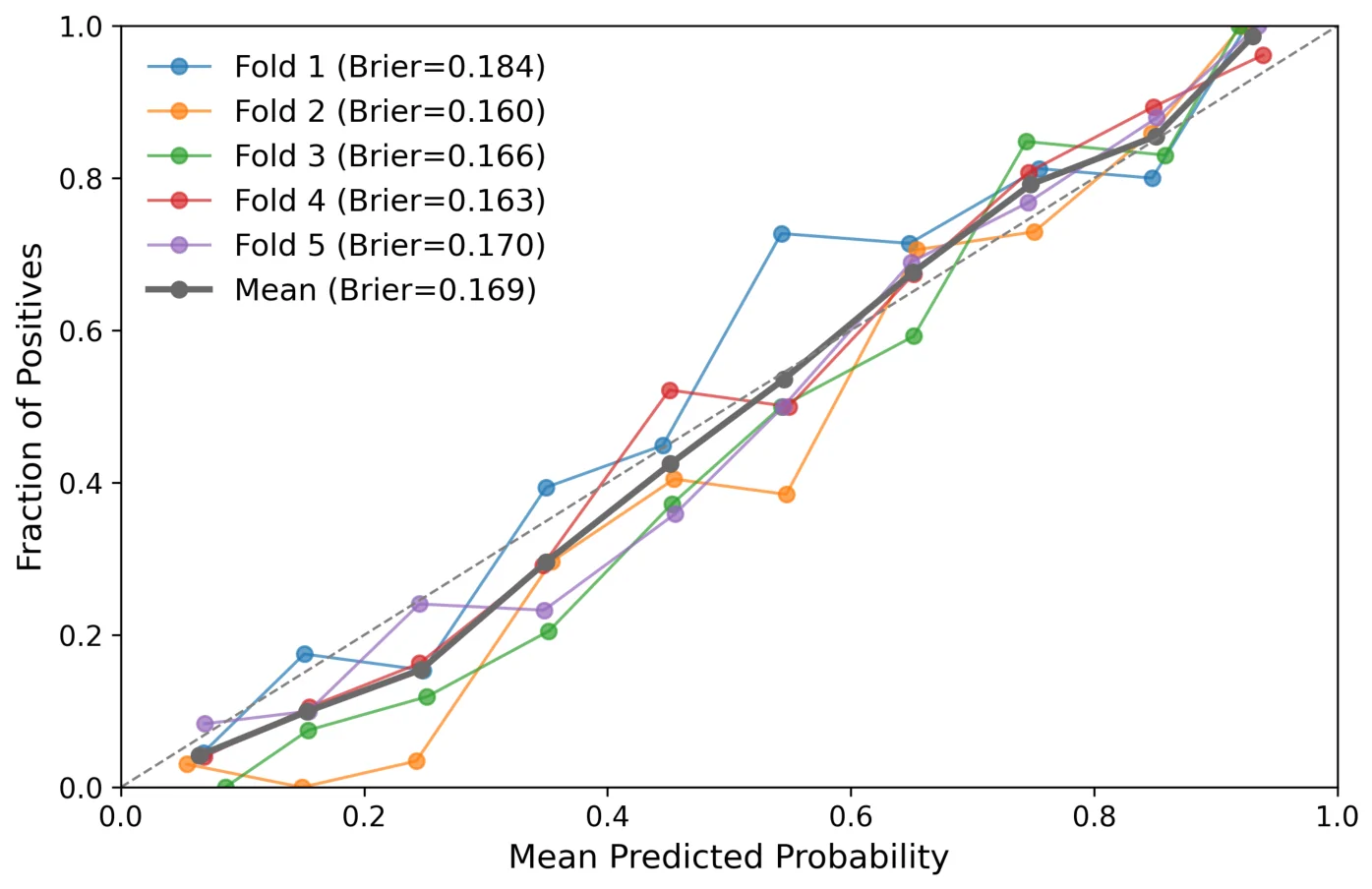
Calibration curves of the hybrid fusion model.

As shown by the calibration curve in Figure 8, the Brier score was 0.169. Although some variability across folds was observed in the low predicted probability range, the mean calibration curve tended to approach the ideal diagonal reference line as the predicted probability increased. These results suggest that the probability estimates of the hybrid fusion model were generally well calibrated and that, particularly in the high predicted probability range, the agreement between the predicted probabilities and the observed positive rates was relatively stable.

Overall, the hybrid fusion model showed better discriminative performance and a lower false positive rate than the unimodal models and the multimodal models using a single fusion strategy. These results indicate that ECG signals and quantitative measurements provide complementary information by representing the morphological and the quantitative characteristics of the waveform, respectively, and that combining different fusion strategies allows this information to be exploited more effectively.

#### 4.4.2. Grad-CAM Analysis

**Figure 9:**
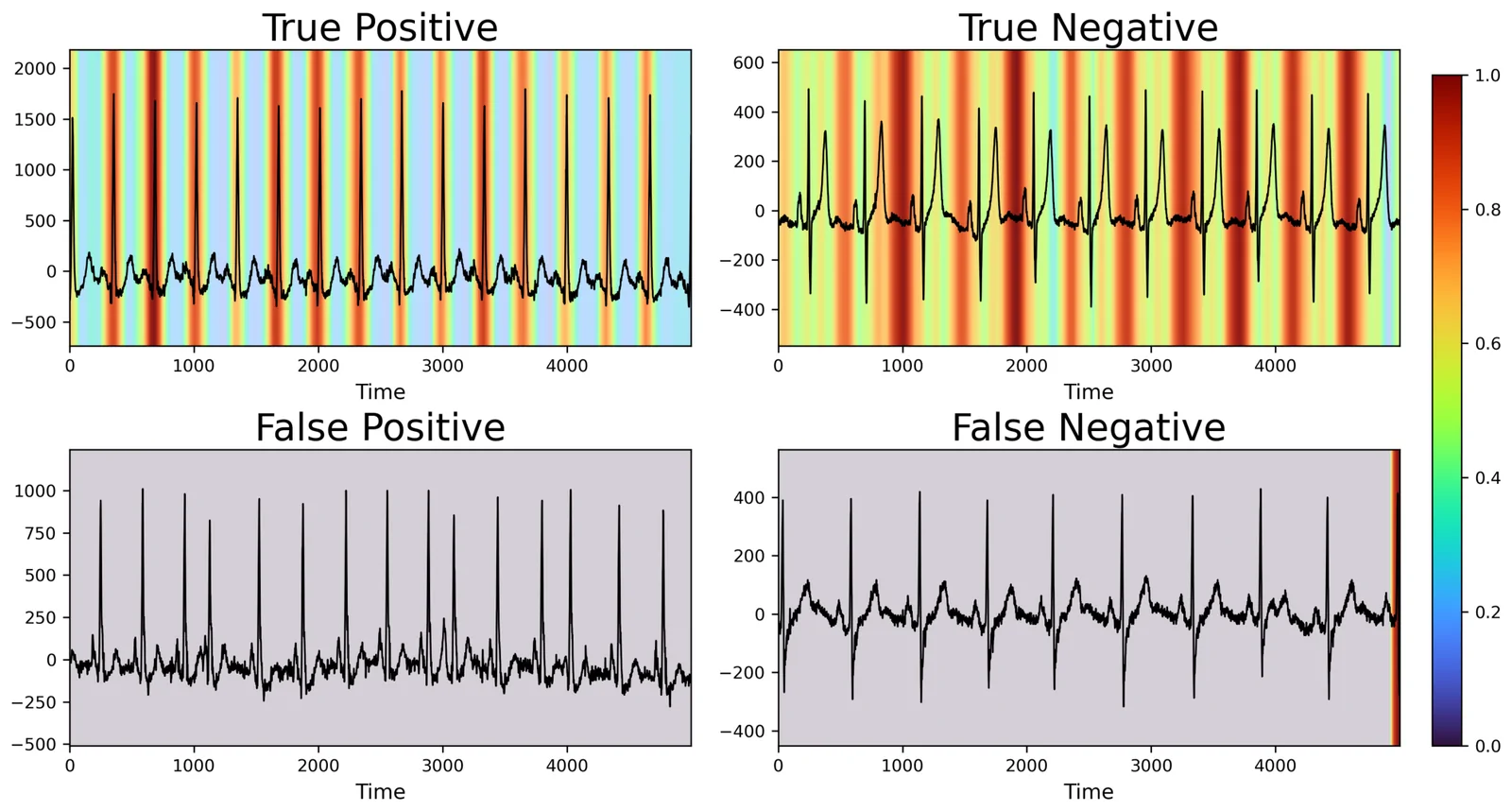
Representative lead II Grad-CAM heatmaps by prediction group.

Grad-CAM was applied to the signal encoder of the interaction early fusion model to analyze the ECG signal segments that the model used most in the prediction process. In the heatmaps, warm colors indicate segments with a relatively high contribution and cool colors segments with a low contribution. Figure 9 shows lead II Grad-CAM heatmaps for representative TP, TN, FP, and FN records. In the correctly classified TP and TN cases, periodic activation bands coinciding with the cardiac cycle were clearly observed. The activation patterns of the two cases nevertheless differed. In the TP case, segments of high and low activation alternated distinctly, whereas in the TN case the overall activation level remained higher and was distributed relatively continuously across the entire signal. This suggests that the model attended selectively to specific waveform segments when predicting positive cases, whereas it may have used the overall regularity of the recording when predicting negative cases.

In the misclassified FP and FN cases, by contrast, little distinct activation structure was observed. Comparing the ECG waveforms of the two cases, the FP case was morphologically similar to the TP case and the FN case to the TN case. This suggests that when the ECG waveforms of the stroke and non-stroke groups appear morphologically similar, the model may not sufficiently capture the morphological evidence required to distinguish the two groups.

**Figure 10:**
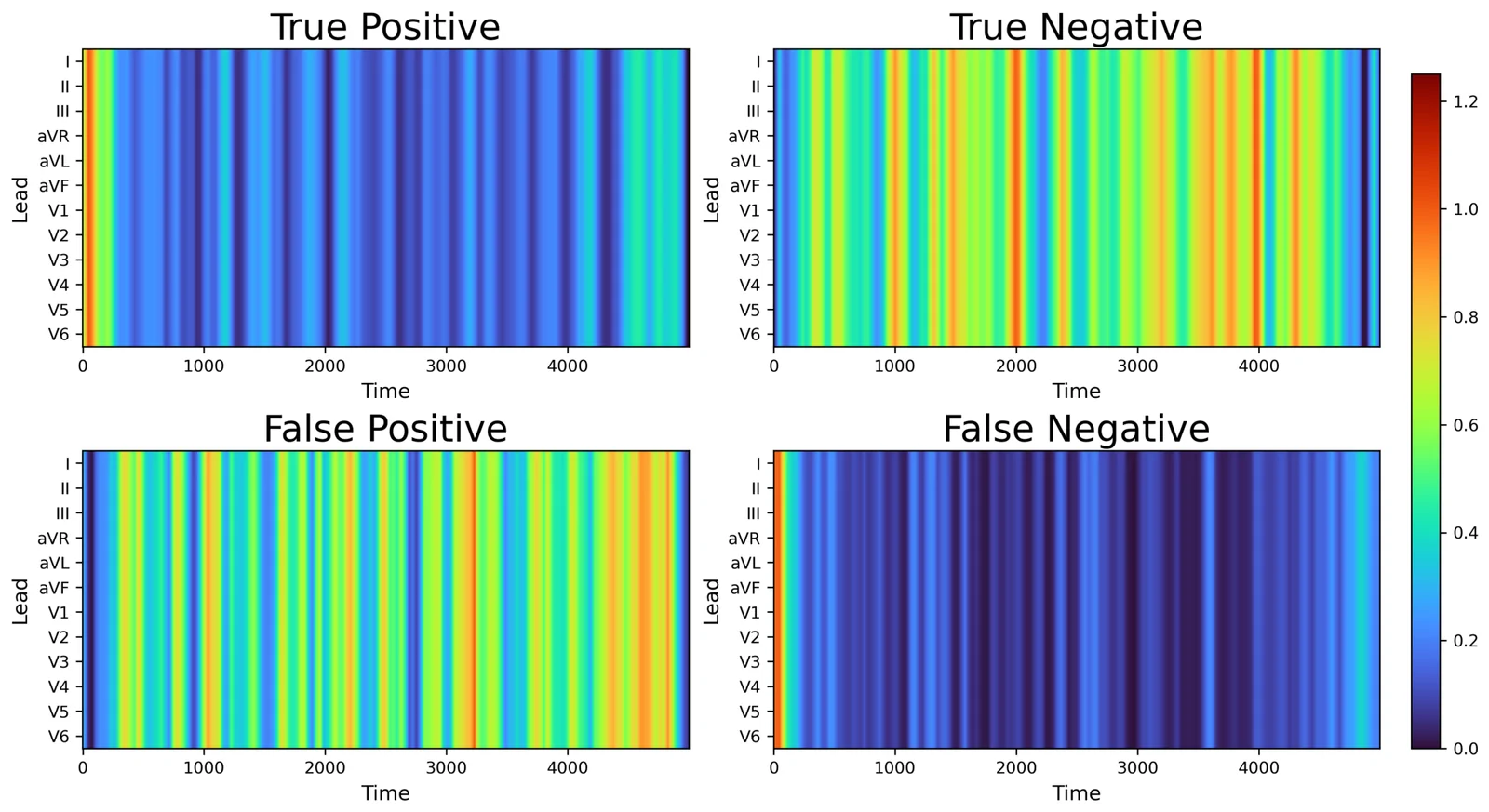
Average Grad-CAM heatmaps by prediction group.

Figure 10 shows the average Grad-CAM heatmaps by prediction group for all 12 leads of the 10 s ECG records. In the TN and FP groups, relatively high activation was broadly distributed across all leads, and periodic vertical activation bands were observed along the time axis. In the TP and FN groups, in contrast, the overall activation intensity was markedly lower. The FN group in particular showed the lowest activation level among the four groups.

Notably, the mean activation intensity was distinguished by the true class label rather than by the correctness of the prediction. The TN and FP groups, which correspond to actual non-stroke patients, showed high activation regardless of the prediction outcome, whereas the TP and FN groups, which correspond to actual stroke patients, both showed low activation. This suggests that the model captured waveform characteristics usable as evidence relatively more clearly in non-stroke cases, whereas the local evidence supporting its predictions was relatively weaker in stroke cases.

Overall, the Grad-CAM analysis shows that the model formed structured activation patterns linked to the cardiac cycle in correctly classified cases, whereas such patterns were not evident in misclassified cases. Grad-CAM, however, is a qualitative method that indicates the signal segments the model used relatively more in the prediction process, so the observed activation regions should not be interpreted directly as clinical biomarkers specific to stroke.

#### 4.4.3. SHAP Analysis

**Figure 11:**
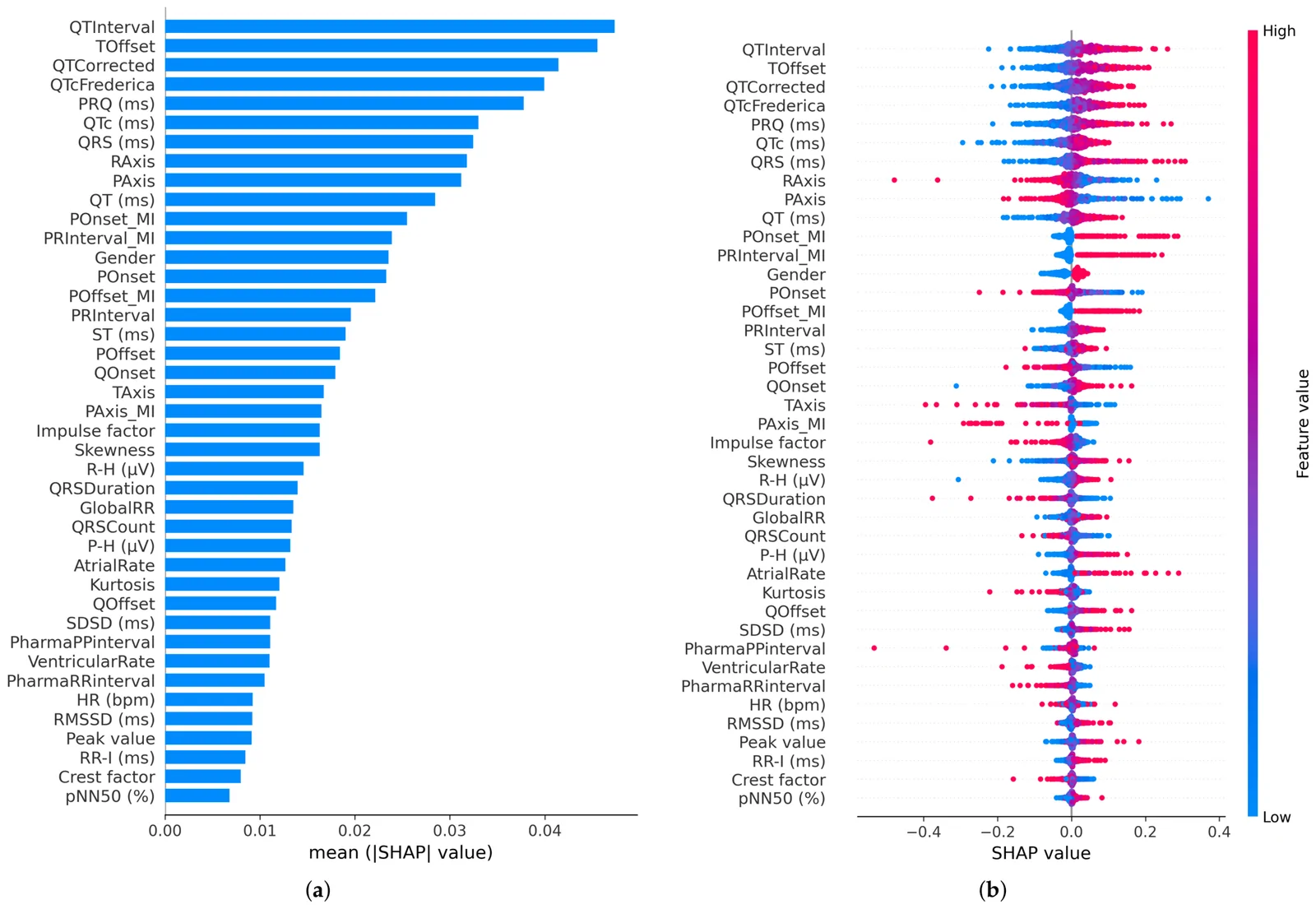
SHAP-based feature interpretation across five folds: (**a**) mean SHAP feature importance; (**b**) pooled SHAP beeswarm plot.

A SHAP analysis was performed on the feature encoder of the interaction early fusion model. Figure 11a shows the feature importance, which indicates the contribution of each feature to the prediction. It was computed by averaging, across folds, the mean absolute SHAP values obtained in each of the five folds. The four most important features were QTInterval, TOffset, QTCorrected, and QTcFrederica, showing that indices related to the QT interval and the T-wave offset had the greatest influence on the decisions of the model. They were followed by PRQ and QTc from the Extracted group, together with QRS, RAxis, and PAxis. QRSDuration from the MUSE group, in contrast, ranked 25th among all 41 variables, a large difference from QRS in the Extracted group, which ranked 7th. The fact that the two variables differed in importance even though both represent the QRS duration shows, consistent with the correlation analysis in Section 4.3.3, that variables representing the same physiological concept can provide different information depending on how they are computed. In addition, the clinical variable Gender ranked 13th, which is consistent with the fact that some ECG measurements, including the QT interval, differ systematically by sex.

The SHAP beeswarm plot in Figure 11b shows the direction and distribution of the influence of each feature value on the model predictions. The SHAP values computed on the test set of each fold were pooled for visualization. The SHAP value on the *x*-axis indicates the direction and magnitude of the contribution to the prediction; positive values contribute to predicting the stroke group and negative values to predicting the non-stroke group. Each point represents an individual record, and the color indicates the magnitude of the feature value, with red denoting high values and blue low values.

The top QT-related features, including QTInterval, TOffset, QTCorrected, and QTcFrederica, showed a consistent pattern of positive SHAP values for high feature values and negative SHAP values for low feature values. In other words, longer QT intervals contributed to predicting stroke. This direction is consistent with the univariate analysis in Table 2, in which the QT and QTc values of the stroke group were significantly longer than those of the non-stroke group, and with previous clinical studies reporting that QTc prolongation is frequently observed in stroke patients [8,9]. Electrical axis-related variables such as RAxis, PAxis, and TAxis, in contrast, showed the opposite direction, with higher values associated with negative SHAP values.

The missingness indicator variables also showed relatively high contributions. POnset_MI, PRInterval_MI, and POffset_MI ranked 11th, 12th, and 15th, respectively, and all three were clearly distributed toward positive SHAP values when the corresponding value was missing. This means that the model shifted its prediction toward stroke when P-wave-related measurements were missing, which is consistent with the missingness analysis in Section 4.3.1, where the missing rates of these variables were significantly higher in the stroke group. PAxis_MI, in contrast, was distributed toward negative SHAP values when missing, showing the opposite pattern; this was the only P-wave-related variable for which the difference in missing rate between the two groups, 0.003 in Table 7, was not statistically significant. These results show that the model used missingness patterns differently according to their association with the class label. Overall, the SHAP analysis shows that the feature encoder based its predictions primarily on QT- and T-wave-related indices, together with P-wave-related missingness patterns, and that the direction of their contributions is consistent with the univariate statistical results and with previous clinical studies.

#### 4.4.4. Error Analysis of Misclassified Cases

To identify characteristics shared by the misclassified cases, the age and the ECG-related variables of the TP, TN, FP and FN groups were compared in an exploratory analysis. To assess the characteristics of misclassification within groups sharing the same true label, false positives were compared with true negatives and false negatives with true positives. Detailed information on cardiovascular comorbidities was not available, so the analysis focused on age, heart rate, QTc and the rate of P-wave measurement failure; the results are presented in Table 13.

The mean predicted probability was 0.636 for the false positives and 0.358 for the false negatives, in both cases closer to the decision threshold than the corresponding correctly classified group (0.731 for the true positives and 0.255 for the true negatives). This indicates that misclassification was concentrated near the threshold rather than among the cases the model predicted with high confidence.

The false positives showed a higher age and a longer QTc than the true negatives. The mean age of the false positives was 67.2 years, compared with 56.8 years for the true negatives, and their mean QTc was 446.9 ms, compared with 438.6 ms. These values were relatively close to the mean age of 70.4 years and the mean QTc of 448.6 ms observed in the true positives. This suggests that some non-stroke records were classified as positive because they shared characteristics with the stroke group in terms of age and QTc. Heart rate, in contrast, was similar in the false positives and the true negatives, at 76.3 and 76.9 bpm, respectively, and the rate of P-wave measurement failure also differed little, at 13.2% and 11.1%.

The false negatives were comparatively younger than the true positives, had a shorter QTc, and showed a lower rate of P-wave measurement failure. The mean age of the false negatives was 65.2 years, compared with 70.4 years for the true positives, and their mean QTc was 442.6 ms, compared with 448.6 ms. The rate of P-wave measurement failure was 10.8% in the false negatives and 19.3% in the true positives. Heart rate, in contrast, was almost identical, at 76.1 and 75.8 bpm, respectively. The difference in the rate of P-wave measurement failure suggests that stroke records accompanied by P-wave abnormalities were captured relatively well, whereas records in which the P wave was measured normally tended to be classified as negative. This shows that a record may be classified as negative even when it belongs to a stroke patient, if the age and ECG characteristics associated with the predictions of the model are comparatively less pronounced.

Taken together, the false positives resembled the true positives in terms of age and QTc, whereas in the false negatives these characteristics were less pronounced than in the true positives. This suggests that the model output is statistically associated with the age and cardiovascular characteristics that accompany stroke in this population, and that false positives may arise in non-stroke records in which such characteristics are present for other reasons. This analysis is, however, an exploratory comparison based on a limited set of variables, and does not include information on cardiovascular comorbidities, medication, or stroke subtype. Confirming the causes of the observed misclassification patterns would therefore require further validation in external data containing more detailed clinical information.

## 5. Ablation Study

### 5.1. Effect of Embedding Size Configuration

In the multimodal framework proposed in this study, the embedding dimensions of the signal encoder, the feature encoder, and the raw interaction branch were set asymmetrically to 512, 128, and 128, respectively. This design is based on the assumption that the raw 12-lead ECG signal contains richer morphological and temporal information than the quantitative measurements and therefore requires greater representational capacity to preserve that information. To assess the validity of this asymmetric embedding design, the proposed configuration (512, 128, 128) was compared with a uniform configuration (256, 256, 256) that assigns the same dimension to all three branches. The embedding dimension configuration applies only within the interaction early fusion model; the architectures of the signal-only and feature-only models are identical under both configurations.

Table 14 compares the performance of the two models under the different embedding dimension configurations. For the interaction early fusion model, the two configurations yielded the same AUC of 0.815, and the Brier scores of 0.179 and 0.178 were essentially identical. Some changes were observed in the threshold-dependent metrics: under the uniform configuration, recall decreased from 80.2% to 77.2% and the F1-score decreased slightly from 75.8% to 75.1%, whereas precision increased from 72.1% to 73.3%. This shows that the embedding configuration affected the operating point of the predictions rather than the overall discriminative ability or the quality of the probability estimates of this model. For the hybrid fusion model, in contrast, the uniform configuration degraded performance on all metrics. Accuracy decreased from 75.5% to 72.6%, a difference of 2.9 percentage points, and the F1-score from 77.6% to 74.9%, a difference of 2.7 percentage points; the AUC decreased from 0.832 to 0.802 and the Brier score increased from 0.169 to 0.199.

Notably, although the standalone performance of the interaction early fusion model was essentially unchanged when the embedding configuration was altered, the performance of the hybrid fusion model containing it degraded markedly. This shows that the embedding configuration of each branch affected how that branch contributed during fusion rather than its standalone discriminative performance. These results indicate that the embedding dimension of each branch is not a matter to be determined solely by the standalone performance of that branch. The (512, 128, 128) configuration, which reflects the relative information content and functional role of each modality, is therefore judged to be a more appropriate design for the final hybrid fusion model than a simple uniform allocation.

In summary, allocating a larger embedding dimension to the raw signal did not change the standalone performance of the interaction early fusion model (AUC 0.815 under both configurations), but the uniform configuration reduced the AUC of the hybrid fusion model from 0.832 to 0.802 and increased its Brier score from 0.169 to 0.199.

### 5.2. Contribution of Each Encoder Branch

Table 15 shows the results of the ablation experiments performed to evaluate the contributions of the three encoders that constitute the interaction early fusion model: the signal encoder, the feature encoder, and the raw interaction branch. In the Encoder Only experiments, the signal encoder showed the best standalone performance among the three encoders, with an AUC of 0.798 and a Brier score of 0.194. The feature encoder was the lowest, with an AUC of 0.671, and the raw interaction branch achieved an AUC of 0.720. Although the raw interaction branch uses both the ECG signal and the quantitative measurements as input, it performed worse than the signal encoder, which uses the signal alone. This may be because some detailed morphological information of the signal was lost when the time dimension was reduced from 5000 to 200 for the self-attention operations. In other words, this branch provides a representation reconstructed from the perspective of inter-modality relationships rather than preserving the signal information as such.

In the Without Encoder experiments, excluding the signal encoder produced the largest performance drop, with the AUC decreasing from 0.815 to 0.659. This shows that the raw ECG signal served as the key information source underpinning the performance of the overall model. Removing the feature encoder and the raw interaction branch, in contrast, yielded AUCs of 0.808 and 0.813, respectively, which differed little from that of the full model and had overlapping confidence intervals. Because the three encoders were trained jointly within a single model, however, their representations may have been adjusted to one another during training. In that case, the performance change observed when a particular encoder is removed does not directly reflect the unique value of the information that encoder provides, but also includes the possibility that the remaining encoders already represent similar information. The performance change observed when encoders are removed one at a time therefore reflects the extent to which the information held by an encoder is also held by the others, rather than how important that encoder is.

This interpretation is consistent with the architecture of the raw interaction branch. Because this branch receives both the ECG signal and the quantitative measurements and represents the interaction between the two modalities, its representation may overlap substantially with that of the signal encoder when the latter is already present. The raw interaction branch thus serves less to add a fully independent information source than to re-express existing information from the perspective of inter-modality relationships.

The contribution of the interaction early fusion branch was more evident at the hybrid fusion stage than within the model itself. In Table 12, the late fusion model, which combines only the predictions of the signal-only and feature-only models, achieved an AUC of 0.818, whereas the hybrid fusion model, which additionally includes the interaction early fusion branch, achieved an AUC of 0.832. This suggests that the branch contributed by providing information distinct from that of the other branches during prediction-level fusion rather than by improving discriminative performance within a single model. Overall, the proposed interaction early fusion model derives its discriminative performance primarily from the morphological information provided by the ECG signal, while the feature encoder and the raw interaction branch can be interpreted as representing that information jointly from different perspectives.

### 5.3. Effect of the Signal Encoder on Performance and Computational Cost

As reported in Section 4.2.1, the difference in standalone classification performance between the two 2D models was small. In the proposed framework, however, the signal encoder is instantiated in both the signal model branch and the interaction early fusion branch, so a comparison at the level of the standalone model does not necessarily reflect its effect at the level of the complete framework. To examine this, a configuration in which the signal encoder was replaced by the 2D ResNet-18 was trained and compared with the EfficientNet-B0 configuration. The signal model branch reused the corresponding model from Table 3, the feature model branch was identical in both configurations, and only the interaction early fusion branch was trained separately with each encoder.

#### 5.3.1. Classification Performance

As shown in Table 16, the AUC improved with each successive stage of fusion for both signal encoders, indicating that the benefit of the proposed fusion strategy is not confined to a particular encoder. The magnitude of the improvement was nevertheless larger for the EfficientNet-B0 configuration. At the signal-only stage the difference in AUC between the two encoders was only 0.005, whereas it widened to 0.014 and 0.012 for interaction early fusion and hybrid fusion, respectively. The increase in AUC from signal-only to hybrid fusion was also larger for EfficientNet-B0, at 0.034 against 0.027 for the 2D ResNet-18. These results suggest that the signal representation learned by EfficientNet-B0 may be exploited more effectively when combined with the measurement-based features.

For the final hybrid fusion model, the EfficientNet-B0 configuration also performed better on the main metrics with the exception of precision. Recall was 8.8 percentage points higher, and at a fixed sensitivity of 90% the false positive rate was 5.3 percentage points lower. This indicates that the EfficientNet-B0 configuration detected more stroke cases while producing comparatively fewer unnecessary positive predictions under a high-sensitivity operating condition. Precision, however, was 2.9 percentage points lower than for the 2D ResNet-18 configuration, so the EfficientNet-B0 configuration was not superior on every operating metric. It nevertheless achieved both a higher AUC and a lower Brier score, and was therefore better aligned with the selection criterion adopted in this study, which prioritizes overall discriminative ability and the quality of the probability estimates.

#### 5.3.2. Computational Complexity and Practical Feasibility

Table 17 reports the computational cost of the framework under the two signal encoders. Because the signal encoder is instantiated in both the signal-only and the interaction early fusion branch of hybrid fusion, the difference in parameter count between the encoders is amplified at the level of the complete framework. The EfficientNet-B0 configuration contains 9.16 M parameters, approximately 60% fewer than the 23.09 M of the 2D ResNet-18 configuration, and fewer even than the 11.17 M of the 2D ResNet-18 signal-only model alone.

In terms of actual computational resources, by contrast, the 2D ResNet-18 configuration was more efficient. Its training time was 8.17 min against 11.80 min for the EfficientNet-B0 configuration, its peak GPU memory was 2288 MB against 8449 MB, and its single-record inference latency was approximately 4 ms against approximately 11 ms. This is because the MBConv blocks of EfficientNet-B0 reduce the parameter count effectively, whereas the intermediate activations produced during channel expansion, together with the efficiency with which these operations map onto the hardware, can increase actual memory use and processing time. The EfficientNet-B0 configuration is therefore advantageous where model size matters, whereas the 2D ResNet-18 configuration may serve as a practical alternative where GPU memory or inference latency is the main constraint.

For the proposed framework, the EfficientNet-B0 based hybrid fusion uses 9.16 M parameters, and the training time summed across its branches was 11.80 min. Because hybrid fusion averages the logits of the three trained branches, the final fusion stage requires no additional trainable parameters and no separate optimization. Peak GPU memory was 8449 MB, which is within the capacity of a single GPU, and inference latency was approximately 11 ms for a single record and 1.24 ms per record at a batch size of 64. Despite the additional cost of the multi-branch design, the proposed framework therefore operates with fewer than 10 M parameters and modest memory and inference requirements, and is computationally practical in a typical single-GPU research environment.

### 5.4. Effect of the Logit Combination Strategy

#### 5.4.1. Branch Logit Distribution and Influence

To examine whether a particular branch dominated the combined result under equal-weight logit averaging, the distribution of each branch logit and its share of the variation in the final combined logit were analyzed. This analysis used the logits produced by the already trained branches, and no branch was retrained.

As shown in Table 18, the mean logit of the three branches ranged from −0.017 to 0.250 and was close to zero in every case, indicating that no branch produced outputs consistently skewed toward a particular class. The standard deviations, in contrast, were similar for the signal-only and interaction early fusion branches at 1.838 and 1.712, respectively, whereas that of the feature-only branch was comparatively small at 0.981. This shows that even when equal weights are applied to the three branches, the effective influence of each branch on the final prediction can differ according to the dispersion of its logits.

The share of the variation in the final logit attributable to each branch was computed from the covariance between the branch logit and the sum of the three logits. The resulting values were 0.466 for signal-only, 0.400 for interaction early fusion, and 0.134 for feature-only. The influence of the feature-only branch, which had the lowest standalone performance, was the smallest; the share of the signal-only branch, the largest of the three, did not exceed one half of the total, however, and the interaction early fusion branch accounted for a comparable share. Differences in the scale and distribution of the logits between branches were therefore present, but no single branch could be regarded as dominating the final logit of the hybrid fusion model.

Equal weighting nevertheless did not guarantee an equal effective influence across the three branches. To examine whether the combination strategy itself affects performance, equal-weight logit averaging was compared with probability averaging and with combinations based on learned weights in the following subsection.

#### 5.4.2. Comparison of Combination Strategies

To assess the appropriateness of equal-weight logit averaging, four strategies differing in combination space and weighting scheme were compared. Strategies with learned weighting were fitted using the validation records of each split only, and the test records were not used at any stage.

As shown in Table 19, the proposed equal-weight logit averaging achieved an AUC of 0.832 and a Brier score of 0.169. Equal-weight probability averaging achieved the same AUC to the reported precision, with a Brier score of 0.172. Performing the combination in probability space therefore did not materially change discriminative performance.

Learning the combination weights on the validation set produced no further improvement. The AUCs of learned logit combination and learned probability combination were 0.829 and 0.828, respectively, both below that of equal-weight logit averaging. This is consistent with the overfitting that may arise when combination weights are additionally estimated from the limited validation data available, as described in Section 3.3.3. The F1-score of learned probability combination was the highest at 77.7%, but the difference from the proposed strategy was only 0.1 percentage points, whereas its accuracy and AUC were lower and its Brier score was higher.

Changing the combination space or learning the weights therefore did not provide a consistent benefit. Equal-weight logit averaging, which requires no additional trainable parameters and showed the most stable performance overall, was retained as the final combination strategy.

### 5.5. Impact of Threshold Selection on Clinical Applicability

Table 20 shows how the performance of the final hybrid fusion model changes when the thresholding strategy is varied to reflect different clinical objectives. Because the AUC and the Brier score are threshold-independent metrics, they remained unchanged at 0.832 and 0.169, respectively. Clear trade-offs appeared, however, in threshold-dependent metrics such as precision, recall, and the FPR, depending on the threshold selected.

With the default threshold of 0.500, the model achieved the highest precision, 74.4%, and the lowest FPR, 29.5%. This shows that this threshold is an advantageous operating point when the objective is conservative decision-making that minimizes false positives. With the Youden index-based threshold of 0.483, which maximizes the sum of sensitivity and specificity, accuracy was maintained at 75.5% while recall improved to 82.7%. The main results of this study were computed using this threshold.

In acute care settings, where the risk associated with false negatives is particularly high, securing high recall is more important. With the threshold of 0.436 that maximizes the F1-score, recall increased to 87.8% and the F1-score reached 78.5%, the highest among all threshold settings. When the threshold was further adjusted to 0.391 to meet 90% sensitivity, recall increased to 90.5%. Precision, however, decreased to 68.1% and the FPR increased to 45.2%, meaning that approximately 45% of non-stroke patients were classified as positive.

Two points should be considered when interpreting these results. First, the dataset used in this study has an approximately 1:1 ratio of the stroke and non-stroke groups, which differs from the prevalence in real clinical settings. In settings with a lower stroke prevalence, the positive predictive value would be lower than the precision presented in Table 20 even if the same sensitivity and specificity were maintained. Second, the thresholds obtained under each strategy varied considerably across folds. The 95% confidence interval of the Youden-based threshold, for example, ranged from 0.383 to 0.583, a range that includes both the default threshold of 0.500 and the F1-maximizing threshold of 0.436. This indicates that the optimal threshold was not estimated stably as a single value and suggests that separate threshold recalibration using data from the target institution would be required in practice.

Despite these limitations, the results in Table 20 show that the proposed hybrid fusion model is not restricted to a single fixed threshold but can be adjusted to different operating points according to the clinical objective. It can thus accommodate a range of clinical requirements, from conservative decision-making intended to reduce unnecessary follow-up examinations to sensitivity-oriented operation intended to minimize missed high-risk patients.

## 6. Generalization and Statistical Analysis

### 6.1. Statistical Comparison Between Signal-Only and Hybrid Fusion Models

To determine whether the performance improvement of the proposed hybrid fusion model over the signal-only model was statistically significant, additional statistical comparisons were performed according to the characteristics of each evaluation metric. Because the AUC is a discriminative performance metric that does not depend on a specific threshold, the difference in AUC between the two models was evaluated using the DeLong test [64]. Accuracy, precision, recall, the F1-score, and the Brier score were compared using a bootstrap-based test that resamples the pooled out-of-fold predictions at the patient level [65], and a paired *t*-test [66] was additionally performed as a supplementary analysis to examine fold-level performance differences. The results of both tests are presented together in Table 21.

In the DeLong test, the pooled AUC of the hybrid fusion model was 0.830, which was 0.036 higher than the 0.794 of the signal-only model, and this difference was statistically significant (p<0.001). Here, the pooled AUC is not the mean of the AUCs computed in each fold but the value computed from the out-of-fold predictions of all folds combined. A significant difference was also confirmed by the paired *t*-test (p=0.003), and the Brier score was significant in both tests as well (bootstrap p<0.001; paired *t*-test p=0.009).

For the threshold-dependent metrics, the hybrid fusion model improved recall by 0.068 (6.8 percentage points), the F1-score by 0.034 (3.4 percentage points), and accuracy by 0.026 (2.6 percentage points) relative to the signal-only model, and all three showed significant improvements in the bootstrap test (p<0.001, p<0.001, and p=0.002, respectively). Precision, in contrast, increased by only 0.004 (0.4 percentage points), and no significant difference was observed (p=0.572). This can be regarded as a consequence of the operating point of the model shifting toward recall at the Youden index-based threshold; the improvement in false positive control was more evident in the FPR at 90% sensitivity (a decrease from 52.7% to 45.2%) than in precision.

For accuracy, recall, and the F1-score, the two tests yielded different results. All three metrics were significant in the bootstrap test (p=0.002, p<0.001, and p<0.001, respectively) but not in the paired *t*-test (p=0.068, p=0.253, and p=0.155, respectively). This is because the bootstrap test evaluates sampling variability at the level of the entire cohort, whereas the paired *t*-test reflects variability due to the data split. The 95% confidence interval for the recall of the hybrid fusion model was indeed wide, ranging from 74.3% to 91.1%, which is also why the power of the paired *t*-test is low when the number of folds is limited to five. In summary, the improvements in discriminative performance and probability estimation of the proposed hybrid fusion framework were confirmed consistently by both testing approaches, whereas the magnitude of the performance gain at a specific operating point may vary with the composition of the data.

### 6.2. Subgroup Analysis by Sex and Age

Table 22 presents the subgroup evaluation performed to determine whether the proposed final hybrid fusion model maintains consistent performance across demographic subgroups differing in sex and age. In the sex-based analysis, the model achieved AUCs of 0.837 and 0.826 in the male and female groups, respectively, exceeding 0.82 in both groups. The overall performance of the male group (accuracy 76.3%, F1-score 79.3%) was slightly higher than that of the female group (accuracy 74.5%, F1-score 75.1%), and the Brier scores were similar at 0.166 and 0.172, respectively. Considering that the AUC confidence intervals of the two groups overlapped, no clear performance gap by sex was observed in this analysis.

The age-based analysis was performed by dividing the entire cohort into three groups: under 65 years (<65), 65–74 years, and 75 years or older (≥75). The AUCs of the three groups were 0.834, 0.797, and 0.812, respectively, with the under-65 group recording the highest value and the lowest Brier score (0.165). The AUC confidence intervals of the three groups all overlapped, however, and the intervals were particularly wide for the 65–74 group (0.725–0.869) and the ≥75 group (0.729–0.896). This is attributable to the limited number of records in each subgroup: the mean number of records per fold was 96.8 in the 65–74 group and 137.0 in the ≥75 group. It is therefore difficult to draw firm conclusions about differences in discriminative performance across age groups.

In the threshold-dependent metrics, recall increased with age. Recall was 74.5% in the under-65 group, 86.3% in the 65–74 group, and 86.9% in the ≥75 group. These differences, however, may reflect differences in the composition of the subgroups, such as the age distribution and the proportion of stroke cases, rather than a change in the discriminative ability of the model. In terms of the mean number of records per fold, the proportion of stroke cases was 39.6% in the under-65 group but 60.7% in both the 65–74 and the ≥75 groups. As shown in the error analysis in Section 4.4.4, the model assigned higher predicted probabilities in the older groups, and the proportion of stroke cases was also higher in these groups, so recall and precision rose together; precision indeed changed in the same direction as recall, at 68.9%, 74.7%, and 75.9%. In addition, the threshold-independent AUC was in fact slightly lower in the older groups. The higher recall observed in the older groups is therefore difficult to interpret as evidence that the model identifies older patients better. Overall, these subgroup results show that the proposed hybrid fusion framework operated without a clear performance gap despite physiological differences in ECG characteristics by sex and age.

### 6.3. Temporal Robustness and Potential Confounding Analysis

#### 6.3.1. Common-Period Temporal Holdout Evaluation

As described in Section 2.1, the ECG collection periods of the stroke and non-stroke groups differed, so it is necessary to rule out the possibility that the observed classification performance was attributable to the difference in collection period rather than to disease state. To examine this, a separate evaluation was performed using only the common period from 2015 to 2019, during which data from both groups were available. The common-period cohort comprised 3132 ECGs in total, of which 1122 (35.8%) were records of stroke patients. All ECGs of the non-stroke group fell within this period, whereas 1015 records of the stroke group collected in 2011–2014 and 2020–2021 were excluded. The common-period cohort therefore differs from the full cohort in both sample composition and class ratio.

The evaluation was performed using a five-fold holdout scheme based on the acquisition year. As presented in Table 23, one year was designated as the holdout in each fold and the ECGs of the remaining four years were used for training, with the ECGs of the holdout year split equally into a validation set and a test set. This design ensures that the model is evaluated on data from a period not used for training, making it possible to determine whether it relies on patterns specific to a particular collection period. The proportion of stroke cases in the test set of each fold ranged from 32.0% to 40.7%.

Table 24 shows the classification performance on the common-period cohort. The hybrid fusion model recorded an AUC of 0.820, an F1-score of 67.6%, and a Brier score of 0.177, the best performance among the four configurations compared. In particular, the AUC improved by 0.032 over the best unimodal model, signal-only (AUC 0.788), which is almost identical to the improvement of 0.034 observed in the main experiment on the full cohort. The AUC was also 0.020 higher than that of interaction early fusion (0.800), and the Brier score improved from 0.194 for signal-only to 0.177, indicating a concurrent improvement in the accuracy of the probability estimates. The ranking of the models, feature-only (0.694), signal-only (0.788), interaction early fusion (0.800), and hybrid fusion (0.820), was also consistent with Table 12, and the absolute performance of hybrid fusion remained similar to that in the main experiment (AUC 0.832, Brier score 0.169). The performance gain from fusion was maintained even when all stroke records collected outside the common period were excluded and a temporal holdout scheme was used. This suggests that the benefit of the proposed hybrid fusion was not created by the difference in collection period between the two groups.

Clear changes were observed, however, in the threshold-dependent metrics. The precision of the hybrid fusion model decreased from 73.5% to 61.3% and the F1-score from 77.6% to 67.6%. This can be regarded as reflecting the change in the class ratio of the cohort rather than a decline in the discriminative ability of the model. The proportion of stroke cases in the common-period cohort was 35.8%, lower than the 51.5% of the full cohort, and a lower prevalence reduces the positive predictive value, that is, the precision, even at the same sensitivity and specificity. The threshold-independent AUC and Brier score, in fact, changed by only 0.012 and 0.008, respectively. As discussed in Section 5.5, these results demonstrate on real data that threshold-dependent metrics can vary with the prevalence in the deployment setting.

In summary, under the year-based holdout evaluation restricted to the common period from 2015 to 2019, the hybrid fusion model achieved an AUC of 0.820 and a Brier score of 0.177, and its AUC improvement over the signal-only model (0.032) was nearly identical to that observed in the full cohort (0.034). Because the threshold-independent AUC and Brier score changed only slightly, the decreases in precision and F1-score are more likely to reflect the lower proportion of stroke cases in this cohort than a loss of discriminative ability. Restricting the analysis to the common period, however, changed both the cohort composition and the class distribution, so an additional sensitivity analysis intended to assess the influence of collection period on classification performance more directly is presented in Section 6.3.2.

#### 6.3.2. Sensitivity Analysis of Potential Temporal Confounding

The temporal holdout evaluation in Section 6.3.1 controlled for the influence of collection period information during training by retraining the model on common-period data only. Because the cohort composition and the class distribution changed in the process, however, it is necessary to determine more directly how much the collection period itself contributes to distinguishing the two groups. Three additional sensitivity analyses were therefore performed on the model trained on the full-period data, and the results are summarized in Table 25.

First, stroke status was predicted using only the acquisition year for the records in the 2015–2019 common period, during which data from both groups were available. The Year-Only model achieved an AUC of 0.490, close to chance level. This shows that, within the common period, the acquisition year provided almost no information for distinguishing the two groups.

Next, the test set predictions of each fold were evaluated on the full cohort and on the subset restricted to the 2015–2019 common period, and the resulting AUCs were averaged across folds. The AUC of the hybrid fusion model was 0.832 for the full period and 0.821 for the common period, a difference of only 0.011 between the two cohorts. This suggests that the discriminative performance observed over the full period was not driven primarily by the stroke records collected outside the common period.

Finally, to evaluate the relationship between the model scores and the collection period while holding disease state constant, the predicted logits generated by the hybrid fusion model were used to distinguish the common period from the non-common period among stroke patients only. The AUC in this analysis was 0.533, close to chance level and markedly lower than the stroke classification performance (AUC 0.832). This suggests that, among stroke patients, the model output carries little information about the collection period.

In summary, the acquisition year provided almost no discriminative information within the common period, discriminative performance was maintained even when the evaluation cohort was restricted to the common period, and the association between the model scores and the collection period was also close to chance level. Because no non-stroke control group existed in the non-common period, disease effects and temporal effects could not be fully separated. Considering these findings together with the result in Section 6.3.1, where the model retrained on common-period data alone maintained an AUC of 0.820, it is nevertheless unlikely that temporal factors alone explain the classification performance obtained in this study.

## 7. Discussion

### 7.1. Interpretation of Model Performance

This study used information obtained from the same 12-lead ECG record, separated into two different forms of representation: the raw signal and the quantitative measurements. The fact that the signal-only model (AUC 0.798) outperformed the feature-only model (0.718) suggests that the representation obtained from the raw waveform showed a stronger statistical association with the stroke population than the representation obtained from the quantitative measurements. That the hybrid fusion model achieved an AUC 0.034 higher than the signal-only model, with the difference being statistically significant (p<0.001), indicates that the measurements contain structured quantitative information that the raw signal alone does not fully capture. This suggests that complementary effects similar to those of multimodal learning can be obtained from multiple representations of a single data source.

In terms of fusion strategy as well, combining different levels of fusion was more advantageous than using a single strategy. The hybrid fusion model (AUC 0.832) outperformed both the early fusion model (0.810) and the late fusion model (0.818). Removing the raw interaction branch in the encoder ablation changed performance only slightly, which likely reflects redundancy among the jointly trained encoders rather than the absence of a contribution. Indeed, the gain from late fusion to hybrid fusion, which differ only in the interaction early fusion branch, suggests that this branch contributes mainly by providing distinct information at the prediction level. The pretrained ECG foundation models ECG-FM (0.717) and HuBERT-ECG (0.761), on the other hand, performed worse than the signal-only model on the dataset used in this study, although this may be influenced by the dataset size and the fine-tuning configuration.

### 7.2. Interpretation of Feature Characteristics

With respect to the quantitative measurements, not all variables contributed equally to classification performance, and these differences arose from the mechanism generating the missingness, the stability of the estimates, and the redundancy between variables. In terms of the mechanism generating missingness, missingness in QTcFrederica was more closely related to the acquisition year than to disease characteristics, and its missingness indicator was therefore excluded from the final feature set, whereas missingness in the P-wave-related variables may reflect a possible non-random pattern associated with age and disease state. Missingness information should therefore be used selectively according to its generating mechanism.

In terms of estimation stability, a trade-off was apparent. Removing an unstable feature reduces the influence of estimation error and missingness, but useful information may be lost along with it. Robust spectral estimation or aggregation across multiple windows can preserve such information while attenuating the variability, yet it cannot overcome the fundamental limitation imposed by the short observation time and the small number of RR intervals available in a 10 s ECG. HF reflects comparatively short cycles and therefore leaves some room for robust estimation, but the short-term heart rate variability it represents may be partly captured by RMSSD, which showed higher estimation stability in the present analysis. Because classification performance improved after the frequency-domain features were removed, we prioritized estimation stability and model simplicity over the preservation of information and removed them.

In terms of redundancy between variables, the configurations that removed variables highly correlated across the two feature groups did not perform best, and the outcome depended on which group of variables was removed. Overall, feature selection is better understood as a process that jointly considers the missingness mechanism, estimation stability, and redundancy of each variable than as a process of removing redundant or unstable variables.

### 7.3. Model Interpretability

The Grad-CAM analysis showed that the signal encoder used a variety of time segments rather than consistently focusing on a single waveform component. No stereotyped waveform pattern was observed even among cases sharing the same true label, and in the group-average analysis the TP and FN groups showed similarly low activation and the TN and FP groups similarly high activation despite their different prediction outcomes. This suggests that stroke and non-stroke ECGs do not each have a single fixed morphology and that the final prediction may have been determined by the temporal location of the activation and its integration with the feature encoder information rather than by the overall magnitude of the activation. In particular, because the non-stroke group in this study consisted of patients admitted for various reasons rather than healthy controls, ECG abnormalities may be observed in both groups, and this morphological overlap may have constrained the learning of consistent classification criteria from the raw signal alone.

In the SHAP analysis, QT- and T-wave-related variables ranked highest, and longer repolarization intervals contributed toward stroke, consistent with the univariate analysis in Table 2 and with previous studies [8,9]. The P-wave-related missingness indicators also contributed toward stroke when missing, in line with their higher missing rates in the stroke group. Because SHAP values represent contributions conditional on the other input variables, however, the contribution direction of an individual variable should not be interpreted as causal evidence.

### 7.4. Clinical Implications

A 12-lead ECG can be acquired rapidly and non-invasively, and electrocardiographic examination is recommended in the initial evaluation of patients with acute ischemic stroke [67]. A model that produces an ECG-based classification score therefore has potential as an auxiliary decision-support tool for identifying patients who require further evaluation.

The non-stroke control group consisted of inpatients without a stroke diagnosis and was not restricted to patients presenting with acute neurological symptoms resembling stroke. This composition provides proof-of-concept evidence for the ability to distinguish stroke patients from patients with diverse clinical backgrounds. Real screening situations, however, may include conditions that mimic stroke symptoms, such as seizures, syncope, toxic and metabolic disturbances, and peripheral vertigo [68,69], and a different disease spectrum in the control group may alter sensitivity and specificity through the spectrum effect [70]. Moreover, because the positive predictive value depends on the prevalence in the target population [71], the performance observed in this study should be validated further in a symptom-matched cohort including stroke mimics rather than extrapolated directly to real screening settings. In addition, securing 90% sensitivity results in a false positive rate of 45.2%, and threshold recalibration using data from the target institution would be required for actual deployment.

If validated in external and prospective studies, the proposed approach may also offer several potential benefits. First, because a 12-lead ECG can be acquired relatively easily even in small hospitals and in the prehospital setting, the classification score could help identify, at an early stage, patients who require further evaluation or transfer in environments where immediate access to neuroimaging or neurologists is limited. Second, because ECGs are recorded repeatedly in hospitalized patients for other purposes, automatically computing the score whenever a record is generated in the ECG management system could help flag patients with suspected in-hospital stroke, although this setting was not evaluated in the present study. Third, because the framework operates on the ECG record alone without linkage to electronic medical records or other data sources, it may be easier to evaluate across institutions, for example through multicenter validation and federated learning. Fourth, the approach of integrating the raw signal and the device-derived measurements of the same record at multiple levels is not specific to stroke and could be extended to other ECG-based classification tasks or to other biosignals for which devices also provide quantitative measurements.

### 7.5. Limitations and Future Directions

This study is based on data collected retrospectively at a single institution, so it has not been confirmed whether the same performance is reproduced at other institutions or in other equipment environments. In the subgroup analyses by sex and age, the confidence intervals overlapped substantially and no clear performance differences were observed, but the sample sizes of the subgroups were uneven, and the influence of ethnicity, comorbidities, and sociodemographic factors could not be evaluated.

In addition, the data collection periods of the stroke and non-stroke groups did not overlap completely. The sensitivity analyses in Section 6.3 consistently suggested that temporal factors alone are unlikely to explain the classification performance. Because no non-stroke control group existed in the non-common period, however, disease effects and temporal effects could not be fully separated.

The input data also have limitations. Minimizing the use of clinical information has the advantage of simplifying the conditions for application, but clinical context such as symptom onset time, neurological severity, cardiovascular comorbidities, medication history, and laboratory findings was not reflected. In addition, because stroke subtype information was unavailable, subtype-specific performance could not be evaluated.

A further limitation concerns the interpretation of the missingness indicators. We analyzed the association between these indicators and acquisition-related factors, excluded the indicator that depended strongly on acquisition year, and examined the predictive contribution of the remaining indicators through feature removal experiments. The training procedure, however, imposes no explicit constraint that prevents acquisition artifacts from being used. It therefore could not be determined whether the contribution of the retained P-wave indicators arises from waveform characteristics associated with stroke or from signal quality and acquisition or measurement conditions. We do not interpret the contribution of these variables as a stroke-specific signal.

To address these limitations, the following extensions are needed. First, future work should evaluate generalizability using multicenter external datasets in which both groups are collected over the same period. Such datasets would also allow subgroup performance to be examined in cohorts with more diverse ethnic and sociodemographic characteristics. In particular, these studies should include a symptom-matched control group with stroke mimics and collect subtype information so that subtype-specific performance can be evaluated. Second, incrementally integrating clinical information would make it possible to determine both the independent contribution of the ECG-based model and the additional benefit of combining clinical information. Third, to reduce the possibility that the MI variables reflect acquisition conditions, signal quality indices and acquisition-related metadata, such as the recording device and the measurement algorithm, could be collected together, so that performance can be evaluated across these factors or representations invariant to acquisition conditions can be learned. Fourth, if longer ECG recordings or repeated measurements become available, the frequency-domain HRV information excluded in this study because of its low estimation stability could be reintroduced. Finally, approaches that use the signal in a more fine-grained manner, such as lead-wise analysis or beat-level segmentation, are expected to improve both classification performance and interpretability.

## 8. Conclusions

In this study, we proposed an ECG-centric hybrid multimodal classification framework that integrates raw 12-lead ECG signals with quantitative measurements derived from the same record, and evaluated its performance in a real clinical cohort. Through an interaction early fusion architecture containing a raw interaction branch, the proposed framework learns inter-modality relationships at both the raw data level and the representation level and integrates them with the predictions of the signal-only and feature-only models, so that the advantages of early and late fusion are exploited together.

In the experiments, the proposed hybrid fusion model achieved an AUC of 0.832 and a Brier score of 0.169, the highest AUC and lowest Brier score among the evaluated models, including the unimodal models, the pretrained ECG foundation models, and the baseline multimodal models using a single fusion strategy. These findings suggest that two different forms of representation derived from a single ECG record provide complementary information and that a strategy integrating them at multiple levels can contribute to improved stroke classification performance. In addition, this study minimized dependence on ancillary clinical information and used only the standard 10 s 12-lead ECG routinely collected in hospitals. Pending external and prospective validation, the proposed framework could potentially be developed into an auxiliary decision-support tool that identifies patients requiring further evaluation, using ECG characteristics statistically associated with stroke.

This study is nevertheless based on data collected retrospectively at a single institution, and the ECG collection periods of the two groups did not overlap completely. Several sensitivity analyses suggested that temporal factors alone are unlikely to explain the classification performance, although disease and temporal effects could not be fully separated. In addition, it has not been confirmed whether the same performance is reproduced at other institutions and in other patient populations. Future work should confirm generalizability and clinical effectiveness through validation on multicenter external datasets in which both groups are collected over the same period and through prospective evaluation, and should extend the approach to related tasks such as stroke subtype classification.

## Figures and Tables

**Table 1:** Description of the ECG feature sets.

MUSE Measurements (n=19)		Extracted Measurements (n=21)	
Feature Name	Description	Feature Name	Description
VentricularRate	Beat rate of ventricles (bpm)	HR	Heart rate derived from RR intervals (bpm)
AtrialRate	Beat rate of atria (bpm)	RR-I	Mean RR interval (ms)
GlobalRR	Average RR interval across leads (ms)	PRQ	Time from P onset to R peak (ms)
QRSCount	Number of QRS complexes detected	QRS	QRS duration (ms)
PRInterval	Time from P onset to QRS onset (ms)	QT	QT interval (ms)
QRSDuration	Duration of the QRS complex (ms)	QTc	Rate-corrected QT interval
QTInterval	Time from Q onset to T offset (ms)	ST	ST-segment duration (ms)
QTCorrected	Rate-corrected QT interval (Bazett)	P-H	P-wave height (μV)
QTcFrederica	Rate-corrected QT interval (Fridericia)	R-H	R-wave height (μV)
PharmaRRinterval	Pharmacologic RR interval (ms)	RMSSD	Root mean square of successive RR differences
PharmaPPinterval	Pharmacologic PP interval (ms)	SDSD	Standard deviation of successive RR differences
QOnset	Onset of the QRS complex (ms)	pNN50	Proportion of RR differences greater than 50 ms (%)
QOffset	Offset of the QRS complex (ms)	LF	Low-frequency power of HRV (0.04–0.15 Hz)
POnset	Onset time of the P wave (ms)	HF	High-frequency power of HRV (0.15–0.4 Hz)
POffset	Offset time of the P wave (ms)	VLF	Very-low-frequency power (<0.04 Hz)
TOffset	Offset time of the T wave (ms)	LF/HF	Ratio of LF to HF power
PAxis	Electrical axis of the P wave (°)	Kurtosis	Measure of the peakedness of the ECG amplitude
RAxis	Electrical axis of the R wave (°)	Skewness	Asymmetry of the ECG amplitude distribution
TAxis	Electrical axis of the T wave (°)	Peak value	Maximum amplitude of the ECG waveform
		Impulse factor	Ratio of peak amplitude to mean amplitude
		Crest factor	Ratio of peak amplitude to RMS amplitude

Abbreviations: bpm, beats per minute; HRV, heart rate variability; LF, low frequency; HF, high frequency; VLF, very low frequency; RMS, root mean square.

**Table 2:** Statistical results for MUSE and extracted ECG variables.

MUSE Variables (n=25)				Extracted Variables (n=25)			
Variable	Stroke	Non-Stroke	*p*-Value	Variable	Stroke	Non-Stroke	*p*-Value
VentricularRate	75.80±17.84	76.56±18.94	0.186	HR	76.89±17.56	77.54±18.64	0.252
AtrialRate	86.08±52.73	84.27±46.22	0.240	RR-I	817.59±172.27	813.03±172.05	0.395
GlobalRR	831.45±181.80	824.44±177.58	0.210	PRQ	238.57±36.94	232.88±36.15	<0.001
QRSCount	12.45±2.98	12.58±3.14	0.159	QRS	93.80±19.58	90.15±19.20	<0.001
PRInterval	166.46±25.60	162.24±26.05	<0.001	QT	360.78±58.34	348.50±61.08	<0.001
QRSDuration	92.26±15.82	93.54±18.13	0.016	QTc	401.41±51.97	389.18±58.16	<0.001
QTInterval	404.95±44.21	396.39±45.90	<0.001	ST	121.67±43.74	113.88±45.54	<0.001
QTCorrected	446.90±30.82	439.57±39.10	<0.001	P-H	58.59±64.02	52.78±68.27	0.005
QTcFrederica	431.84±28.22	424.29±35.57	<0.001	R-H	56.70±157.74	34.02±174.78	<0.001
PharmaRRinterval	832.09±165.94	829.12±172.38	0.573	RMSSD	139.73±109.23	130.34±113.79	0.007
PharmaPPinterval	812.26±215.77	813.12±208.64	0.897	SDSD	144.39±114.14	134.56±119.68	0.007
QOnset	219.21±6.55	218.60±6.84	0.004	pNN50	49.66±24.94	46.94±24.34	<0.001
QOffset	265.34±8.68	265.37±8.75	0.896	LF	0.00±0.02	0.00±0.10	0.572
POnset	135.91±14.52	137.94±14.38	<0.001	HF	0.01±0.05	0.01±0.14	0.790
POffset	186.12±14.88	188.91±13.89	<0.001	VLF	0.00±0.00	0.00±0.02	0.691
TOffset	421.68±22.80	416.80±23.27	<0.001	LF/HF	0.31±0.20	0.32±0.22	0.388
PAxis	46.19±23.78	49.47±22.99	<0.001	Kurtosis	11.04±7.37	12.45±11.40	<0.001
RAxis	27.38±37.69	37.24±42.51	<0.001	Skewness	1.72±1.56	1.85±1.71	0.010
TAxis	46.44±46.15	48.67±47.99	0.128	Peak value	800.01±375.56	816.72±487.58	0.218
PRInterval_MI	0.15±0.36	0.12±0.32	0.001	Impulse factor	8.42±3.10	8.96±3.55	<0.001
QTcFrederica_MI	0.04±0.21	0.00±0.00	<0.001	Crest factor	5.43±1.34	5.62±1.43	<0.001
POnset_MI	0.15±0.35	0.11±0.31	<0.001	LF_MI	0.16±0.36	0.15±0.36	0.588
POffset_MI	0.15±0.35	0.11±0.31	<0.001	HF_MI	0.16±0.36	0.15±0.36	0.588
PAxis_MI	0.12±0.33	0.12±0.32	0.778	VLF_MI	0.16±0.36	0.15±0.36	0.588
Gender	0.58±0.49	0.55±0.50	0.020	LF/HF_MI	0.16±0.36	0.15±0.36	0.588

Values are presented as mean ± standard deviation. Gender and the MI variables are binary (0/1), so their means correspond to proportions. MI, missingness indicator.

**Table 3:** Performance of ECG signal-based models.

Input	Model	Accuracy	Precision	Recall	F1-Score	AUC	Brier Score
1D	InceptionTime ^1^	67.5 (63.7–71.2)	71.6 (69.7–73.6)	61.5 (46.6–76.3)	65.6 (58.2–72.9)	0.759 (0.730–0.788)	0.201 (0.187–0.215)
	ResNet-18 ^2^	70.7 (68.4–73.1)	69.9 (64.0–75.7)	77.4 (68.5–86.3)	73.1 (71.5–74.6)	0.774 (0.752–0.796)	0.195 (0.184–0.206)
2D	ResNet-18 ^3^	72.6 (69.3–75.9)	71.2 (66.5–76.0)	79.1 (74.1–84.1)	74.8 (72.7–77.0)	0.793 (0.768–0.819)	0.187 (0.174–0.200)
	EfficientNet-B0 ^3^	73.0 (69.5–76.5)	73.0 (69.3–76.7)	76.0 (64.1–87.9)	74.1 (69.0–79.3)	0.798 (0.768–0.827)	0.186 (0.169–0.204)

^1^ Existing implementation; ^2^ 1D implementation based on [13]; ^3^ ImageNet-pretrained 2D implementation. Values are reported as the mean (95% confidence interval) across five folds. Accuracy, precision, recall, and F1-score are expressed as percentages. Higher values indicate better performance, except for the Brier score.

**Table 4:** Classification performance of KNN across the MUSE and extracted feature sets.

Group	Feature Set	*n*	Accuracy	Precision	Recall	F1-Score	AUC	Brier Score
Extracted	LF/HF; ST, QT [3]	3	50.5 (47.2–53.8)	51.7 (49.0–54.4)	41.4 (12.2–70.6)	43.3 (23.7–62.9)	0.523 (0.510–0.535)	0.258 (0.255–0.262)
	Features with *p* < 0.05	3	52.5 (48.8–56.2)	54.6 (50.4–58.7)	53.0 (23.9–82.0)	51.4 (36.4–66.3)	0.539 (0.505–0.573)	0.262 (0.256–0.268)
		5	55.5 (52.0–58.9)	56.2 (53.0–59.5)	61.7 (51.3–72.2)	58.6 (53.8–63.4)	0.568 (0.535–0.601)	0.260 (0.235–0.285)
		7	55.9 (54.3–57.4)	57.5 (54.8–60.3)	55.4 (44.9–66.0)	56.1 (51.0–61.3)	0.576 (0.563–0.589)	0.248 (0.245–0.252)
		10	54.5 (52.7–56.3)	56.1 (52.8–59.4)	59.3 (35.8–82.8)	55.9 (43.3–68.4)	0.566 (0.552–0.581)	0.258 (0.238–0.277)
		12.4	56.3 (52.8–59.8)	57.2 (54.0–60.4)	61.2 (44.7–77.8)	58.5 (50.7–66.3)	0.588 (0.561–0.615)	0.249 (0.245–0.253)
	All	25	55.2 (51.3–59.0)	56.4 (53.5–59.3)	53.5 (34.7–72.3)	54.2 (42.2–66.1)	0.570 (0.550–0.589)	0.251 (0.244–0.257)
MUSE	Features with *p* < 0.05	3	59.3 (56.8–61.7)	58.8 (55.9–61.7)	70.4 (63.6–77.2)	64.0 (61.0–66.9)	0.615 (0.590–0.641)	0.240 (0.231–0.250)
		5	58.5 (55.5–61.6)	59.0 (54.9–63.2)	66.3 (50.8–81.7)	61.7 (55.1–68.3)	0.620 (0.577–0.662)	0.241 (0.228–0.255)
		7	61.2 (58.4–64.0)	61.6 (57.7–65.6)	66.5 (56.5–76.5)	63.7 (59.4–67.9)	0.648 (0.607–0.689)	0.232 (0.222–0.243)
		10	59.9 (56.1–63.6)	59.9 (57.8–61.9)	66.2 (50.1–82.3)	62.4 (54.1–70.7)	0.647 (0.611–0.683)	0.231 (0.222–0.241)
		15.4	58.9 (55.5–62.4)	60.9 (57.7–64.1)	55.9 (46.1–65.6)	58.1 (52.0–64.2)	0.642 (0.612–0.672)	0.235 (0.222–0.248)
	All	25	59.5 (58.5–60.5)	61.5 (56.4–66.7)	61.3 (41.4–81.3)	60.0 (50.9–69.1)	0.664 (0.649–0.679)	0.227 (0.221–0.232)

Features with *p* < 0.05 were selected based on statistical comparisons between the stroke and non-stroke groups within each training fold. The value of n represents the number of features; non-integer values indicate the mean number of features selected across the five folds. Values are reported as the mean (95% confidence interval) across five folds. Accuracy, precision, recall, and F1-score are expressed as percentages.

**Table 5:** Classification performance of MLP across the MUSE and extracted feature sets.

Group	Feature Set	*n*	Accuracy	Precision	Recall	F1-Score	AUC	Brier Score
Extracted	LF/HF; ST, QT [3]	3	52.8 (51.1–54.4)	56.1 (51.6–60.5)	42.6 (26.3–59.0)	47.2 (37.6–56.9)	0.549 (0.522–0.576)	0.249 (0.245–0.252)
	Features with *p* < 0.05	3	54.1 (51.0–57.3)	56.3 (50.6–62.0)	56.5 (35.6–77.5)	55.1 (48.0–62.2)	0.554 (0.522–0.586)	0.248 (0.244–0.252)
		5	55.7 (53.1–58.3)	56.9 (52.7–61.0)	62.3 (48.3–76.3)	58.8 (55.1–62.6)	0.572 (0.551–0.592)	0.247 (0.244–0.249)
		7	55.9 (52.5–59.3)	57.5 (54.1–60.9)	56.2 (36.2–76.3)	55.8 (45.4–66.2)	0.582 (0.566–0.599)	0.245 (0.243–0.248)
		10	57.8 (56.3–59.4)	58.3 (56.6–60.0)	63.7 (56.6–70.8)	60.8 (57.7–63.9)	0.592 (0.576–0.607)	0.245 (0.242–0.248)
		12.4	57.6 (55.5–59.7)	57.5 (55.5–59.5)	68.2 (58.6–77.8)	62.2 (58.2–66.2)	0.601 (0.588–0.614)	0.242 (0.239–0.245)
	All	25	57.8 (54.9–60.6)	57.9 (56.3–59.5)	65.2 (51.2–79.1)	61.0 (54.4–67.6)	0.609 (0.586–0.633)	0.241 (0.237–0.245)
MUSE	Features with *p* < 0.05	3	59.2 (57.5–60.9)	60.0 (57.1–63.0)	62.6 (56.8–68.5)	61.2 (58.9–63.4)	0.630 (0.605–0.654)	0.235 (0.228–0.242)
		5	58.8 (55.8–61.7)	60.2 (55.9–64.6)	61.0 (44.4–77.6)	59.8 (52.0–67.6)	0.637 (0.606–0.668)	0.233 (0.225–0.241)
		7	60.5 (57.3–63.7)	62.3 (60.2–64.3)	58.6 (45.8–71.5)	60.0 (52.1–67.9)	0.652 (0.629–0.674)	0.230 (0.222–0.238)
		10	59.6 (56.7–62.4)	61.7 (59.0–64.4)	56.3 (46.2–66.5)	58.7 (52.2–65.1)	0.645 (0.617–0.673)	0.231 (0.225–0.238)
		15.4	62.6 (59.8–65.4)	62.5 (58.1–67.0)	70.3 (55.7–84.8)	65.6 (60.3–70.8)	0.691 (0.660–0.722)	0.221 (0.211–0.231)
	All	25	64.3 (61.4–67.2)	64.7 (61.3–68.2)	67.9 (59.6–76.2)	66.1 (62.4–69.8)	0.694 (0.656–0.733)	0.220 (0.208–0.233)

Features with p < 0.05 were selected based on statistical comparisons between the stroke and non-stroke groups within each training fold. The value of n represents the number of features; non-integer values indicate the mean number of features selected across the five folds. Values are reported as the mean (95% confidence interval) across five folds. Accuracy, precision, recall, and F1-score are expressed as percentages.

**Table 6:** MLP performance across feature subsets derived from the full 50-feature set.

Feature Set	*n*	Accuracy	Precision	Recall	F1-Score	AUC	Brier Score
Top-10 features	10	60.0 (58.0–61.9)	59.3 (56.9–61.7)	72.1 (62.4–81.7)	64.8 (62.1–67.6)	0.644 (0.626–0.661)	0.232 (0.227–0.238)
Top-20 features	20	59.6 (57.3–61.8)	62.2 (57.5–66.8)	57.2 (46.6–67.8)	59.1 (55.5–62.6)	0.653 (0.643–0.664)	0.232 (0.226–0.238)
Features with p<0.05	27.8	63.2 (59.6–66.7)	65.2 (59.7–70.6)	63.4 (47.8–79.0)	63.5 (57.1–69.9)	0.692 (0.655–0.730)	0.221 (0.209–0.234)
All MUSE + Extracted (p<0.05)	37.4	64.7 (63.2–66.1)	66.8 (65.0–68.6)	62.5 (54.1–70.9)	64.4 (60.5–68.3)	0.703 (0.680–0.726)	0.218 (0.210–0.226)
All features	50	66.3 (65.2–67.5)	66.5 (64.5–68.5)	70.2 (61.4–79.0)	68.1 (64.5–71.7)	0.720 (0.693–0.747)	0.213 (0.204–0.223)

Feature selection based on p<0.05 was performed within each training fold. The value of *n* represents the number of features; non-integer values indicate the mean number of features selected across the five folds. Values are presented as the mean (95% confidence interval) across five folds. Accuracy, precision, recall, and F1-score are expressed as percentages.

**Table 7:** Comparison of feature missing rates between the stroke and non-stroke groups.

Group	Missing Feature	Missing Rate	Stroke	Non-Stroke	Difference	*p*-Value
MUSE	QTcFrederica	0.023	0.045	0.000	0.045	<0.001
	POnset	0.127	0.146	0.108	0.038	<0.001
	POffset	0.127	0.146	0.108	0.038	<0.001
	PRInterval	0.137	0.153	0.119	0.035	0.001
	PAxis	0.119	0.121	0.117	0.003	0.742
Extracted	LF	0.153	0.156	0.150	0.006	0.559
	HF	0.153	0.156	0.150	0.006	0.559
	VLF	0.153	0.156	0.150	0.006	0.559
	LF/HF	0.153	0.156	0.150	0.006	0.559

Missing Rate is the proportion of records in which the corresponding measurement was absent in the full cohort. Difference is the missing rate of the stroke group minus that of the non-stroke group. MI, missingness indicator; HRV, heart rate variability.

**Table 8:** Associations between acquisition-related variables and feature missingness.

Group	Missing Feature	Predictor	Level	OR	*p*-Value	AUC
MUSE	QTcFrederica	Year	Continuous	0.182	<0.001	0.976
	POnset	Age	Continuous	1.059	<0.001	0.715
	POffset	Age	Continuous	1.059	<0.001	0.715
	PRInterval	Age	Continuous	1.058	<0.001	0.712
	PAxis	Age	Continuous	1.057	<0.001	0.712

OR, odds ratio; AUC, area under the receiver operating characteristic curve.

**Table 9:** Within-record variability of the HRV features across overlapping windows of the same recording.

Feature Group	Feature	Valid Records	Median CV [IQR]	CV > 1.0 (%)
Rate	Mean RR	4147	0.013 [0.007–0.026]	0.0
	Heart rate	4147	0.013 [0.007–0.026]	0.0
Time-domain HRV	SDNN	4147	0.191 [0.070–0.600]	9.2
	RMSSD	4147	0.154 [0.064–0.460]	6.4
	SDSD	4147	0.151 [0.065–0.443]	5.8
	pNN50	4054	0.133 [0.071–0.254]	7.1
Frequency-domain HRV	VLF	3367	0.744 [0.441–1.214]	34.9
	LF	3367	0.669 [0.390–1.167]	32.3
	HF	3367	0.565 [0.312–1.010]	25.8
	LF/HF	3367	0.402 [0.223–0.632]	6.5

CV was calculated as the standard deviation divided by the mean of the estimates obtained across overlapping 8 s windows at a 0.5 s stride within each 10 s recording. R peaks were detected once on the full recording, so that the observed variability reflected the sensitivity of the estimates to window position rather than variation in peak detection. Valid records were those yielding at least three usable window-level estimates among the 4147 records. The final column gives the proportion of valid records in which the within-record standard deviation exceeded the corresponding within-record mean. CV, coefficient of variation; IQR, interquartile range; SDNN, standard deviation of NN intervals, which was not included in the model input and is shown as a reference time-domain measure.

**Table 10:** Cross-source feature correlations and their interpretations.

Extracted	MUSE	*r*	Interpretation
HR (bpm)	VentricularRate	0.959	Same heart rate concept
	QRSCount	0.944	Rate-related overlap
	GlobalRR	−0.906	Inverse heart rate–RR relation
	PharmaRRinterval	−0.859	Inverse heart rate–RR relation
RR-I (ms)	GlobalRR	0.959	Same RR interval concept
	VentricularRate	−0.912	Inverse heart rate–RR relation
	PharmaRRinterval	0.907	RR-related overlap
	QRSCount	−0.896	Inverse heart rate–RR relation

*r* denotes the Pearson correlation coefficient computed across all records. Only feature pairs with an absolute correlation of 0.80 or higher between the MUSE and Extracted groups are listed; negative values indicate an inverse relationship.

**Table 11:** Effect of feature group exclusion on classification performance.

Category	Ablation Setting	Input *n*	Accuracy	Precision	Recall	F1-Score	AUC	Brier Score
Baseline	No exclusion	49	64.0 (63.0–65.0)	64.2 (60.9–67.5)	69.3 (57.8–80.8)	66.2 (62.5–70.0)	0.701 (0.686–0.715)	0.220 (0.214–0.226)
MI	MI only	8	50.6 (48.9–52.3)	64.0 (43.8–84.2)	17.3 (3.1–31.5)	24.6 (7.5–41.7)	0.514 (0.473–0.556)	0.248 (0.244–0.252)
	w/o P-wave MI	45	64.0 (60.5–67.5)	64.2 (59.7–68.8)	68.9 (62.4–75.5)	66.3 (63.7–68.9)	0.694 (0.667–0.720)	0.223 (0.212–0.234)
	w/o HRV MI	45	65.2 (62.4–68.1)	64.0 (60.5–67.5)	75.1 (69.4–80.9)	69.0 (67.7–70.2)	0.706 (0.688–0.724)	0.218 (0.212–0.224)
	w/o all MI	41	65.0 (64.0–66.0)	65.1 (61.4–68.9)	70.6 (56.6–84.6)	67.2 (62.4–72.0)	0.709 (0.695–0.724)	0.218 (0.212–0.223)
HRV	w/o HRV set	41	64.9 (62.0–67.9)	64.8 (60.1–69.5)	71.1 (62.8–79.5)	67.5 (65.4–69.7)	0.718 (0.689–0.747)	0.215 (0.204–0.225)
	w/o all MI + HRV set	37	64.8 (61.8–67.8)	64.5 (60.2–68.9)	71.2 (64.4–78.1)	67.5 (65.0–70.1)	0.705 (0.689–0.720)	0.219 (0.212–0.226)
Overlap	w/o MUSE overlap	45	65.5 (62.5–68.4)	64.2 (60.5–67.9)	75.7 (67.9–83.4)	69.3 (67.2–71.3)	0.704 (0.682–0.726)	0.220 (0.212–0.227)
	w/o Extracted overlap	47	64.7 (61.7–67.7)	66.0 (61.4–70.5)	66.5 (54.6–78.5)	65.7 (60.7–70.8)	0.697 (0.673–0.721)	0.221 (0.213–0.229)
	w/o all cross-group overlap	43	64.1 (62.4–65.7)	65.4 (61.3–69.6)	65.0 (58.9–71.1)	65.0 (63.5–66.6)	0.702 (0.680–0.724)	0.220 (0.213–0.227)
Combined	w/o HRV set + MUSE overlap	37	65.3 (64.1–66.6)	66.7 (64.8–68.6)	65.5 (60.6–70.4)	66.0 (64.1–67.9)	0.716 (0.698–0.735)	0.216 (0.208–0.223)

The baseline setting refers to the 49-feature set defined in Section 4.3.1, from which QTcFrederica_MI was excluded because its missingness was strongly associated with the acquisition year; all ablation settings were derived from this baseline. Values are presented as the mean (95% confidence interval) across five folds. Accuracy, precision, recall, and F1-score are expressed as percentages. The column Input *n* indicates the number of input features. MI, missingness indicator; HRV, heart rate variability.

**Table 12:** Comparison of classification performance across unimodal, baseline, and proposed models.

Group	Model	Youden Threshold	Accuracy	Precision	Recall	F1-Score	AUC	Brier Score	FPR at Sens. = 90%
Unimodal	feature-only	0.483 (0.408–0.559)	64.9 (62.0–67.9)	64.8 (60.1–69.5)	71.1 (62.8–79.5)	67.5 (65.4–69.7)	0.718 (0.689–0.747)	0.215 (0.204–0.225)	68.3 (61.2–75.4)
	signal-only	0.488 (0.381–0.595)	73.0 (69.5–76.5)	73.0 (69.3–76.7)	76.0 (64.1–87.9)	74.1 (69.0–79.3)	0.798 (0.768–0.827)	0.186 (0.169–0.204)	52.7 (45.9–59.6)
Baselines	ECG-FM	0.486 (0.439–0.533)	65.4 (61.7–69.2)	65.0 (61.6–68.4)	71.5 (57.7–85.3)	67.7 (61.1–74.3)	0.717 (0.686–0.747)	0.215 (0.204–0.226)	66.3 (58.6–74.0)
	HuBERT-ECG	0.519 (0.389–0.650)	67.5 (65.4–69.5)	68.9 (62.8–75.0)	69.8 (51.7–87.8)	68.3 (62.0–74.6)	0.761 (0.726–0.795)	0.199 (0.185–0.212)	58.1 (52.9–63.3)
	early fusion	0.501 (0.415–0.587)	71.8 (69.4–74.2)	71.9 (66.7–77.1)	75.3 (64.7–85.9)	73.2 (70.0–76.3)	0.810 (0.770–0.849)	0.180 (0.164–0.196)	48.3 (41.7–54.9)
	late fusion	0.448 (0.353–0.542)	73.3 (70.5–76.1)	71.2 (66.0–76.5)	81.9 (75.0–88.8)	75.9 (74.5–77.4)	0.818 (0.789–0.847)	0.176 (0.163–0.190)	47.2 (40.6–53.8)
Proposed	interaction early fusion	0.510 (0.394–0.626)	73.7 (71.7–75.7)	72.1 (68.8–75.4)	80.2 (74.7–85.8)	75.8 (74.3–77.4)	0.815 (0.789–0.841)	0.179 (0.168–0.189)	47.1 (43.7–50.5)
	hybrid fusion	0.483 (0.383–0.583)	75.5 (73.3–77.7)	73.5 (69.3–77.7)	82.7 (74.3–91.1)	77.6 (75.1–80.1)	0.832 (0.809–0.856)	0.169 (0.157–0.181)	45.2 (37.4–53.1)

Values are reported as the mean (95% confidence interval) across five folds. Accuracy, precision, recall, and F1-score were computed at the Youden threshold determined on the validation set of each fold; FPR was computed at the threshold yielding 90% sensitivity. FPR, false positive rate; Sens., sensitivity.

**Table 13:** Characteristics of the prediction outcome groups.

Characteristic	TP	TN	FP	FN
*n*	887	686	325	185
Mean predicted probability	0.731	0.255	0.636	0.358
Age (years)	70.4±12.2	56.8±20.3	67.2±14.8	65.2±14.8
Heart rate (bpm)	75.8±18.0	76.9±19.8	76.3±17.5	76.1±18.2
QTc (ms)	448.6±29.3	438.6±41.8	446.9±33.2	442.6±35.4
P-wave measurement failure (%)	19.3	11.1	13.2	10.8

TP, true positive; TN, true negative; FP, false positive; FN, false negative. The unit of analysis was the test prediction, and the four groups comprised 2083 test predictions generated across the five folds. Continuous variables are reported as mean ± standard deviation. P-wave measurement failure was defined as the absence of one or more of the PR interval, P onset, P offset, and P axis measurements in the MUSE system.

**Table 14:** Performance comparison under different embedding-size configurations.

Model	Embedding Size	Accuracy	Precision	Recall	F1-Score	AUC	Brier Score
Interaction early fusion	(512, 128, 128)	73.7 (71.7–75.7)	72.1 (68.8–75.4)	80.2 (74.7–85.8)	75.8 (74.3–77.4)	0.815 (0.789–0.841)	0.179 (0.168–0.189)
	(256, 256, 256)	73.6 (70.4–76.9)	73.3 (69.8–76.7)	77.2 (71.1–83.2)	75.1 (72.1–78.1)	0.815 (0.782–0.847)	0.178 (0.165–0.191)
Hybrid fusion	(512, 128, 128)	75.5 (73.3–77.7)	73.5 (69.3–77.7)	82.7 (74.3–91.1)	77.6 (75.1–80.1)	0.832 (0.809–0.856)	0.169 (0.157–0.181)
	(256, 256, 256)	72.6 (70.8–74.4)	71.0 (68.3–73.6)	79.4 (76.1–82.7)	74.9 (73.7–76.1)	0.802 (0.775–0.828)	0.199 (0.182–0.217)

Embedding sizes are reported in the order of the signal, feature, and raw-interaction branches. Values are presented as the mean (95% confidence interval) across five folds. Accuracy, precision, recall, and F1-score are expressed as percentages.

**Table 15:** Encoder contribution analysis for the interaction early fusion model.

Setting	Encoder	Accuracy	Precision	Recall	F1-Score	AUC	Brier Score
Interaction early fusion	Full Model	73.7 (71.7–75.7)	72.1 (68.8–75.4)	80.2 (74.7–85.8)	75.8 (74.3–77.4)	0.815 (0.789–0.841)	0.179 (0.168–0.189)
Encoder Only	Signal	72.4 (70.3–74.5)	73.4 (69.3–77.5)	73.4 (65.3–81.6)	73.2 (70.4–75.9)	0.798 (0.775–0.820)	0.194 (0.182–0.207)
	Feature	62.0 (58.6–65.4)	64.2 (59.2–69.3)	59.9 (55.8–63.9)	61.8 (59.4–64.3)	0.671 (0.625–0.716)	0.232 (0.216–0.248)
	Raw Interaction	66.6 (62.9–70.3)	65.2 (61.0–69.4)	76.4 (70.6–82.1)	70.2 (68.0–72.4)	0.720 (0.677–0.762)	0.215 (0.200–0.230)
Without Encoder	Signal	63.2 (59.8–66.6)	62.4 (59.1–65.8)	72.9 (61.2–84.6)	66.9 (62.3–71.6)	0.659 (0.630–0.688)	0.235 (0.231–0.240)
	Feature	72.9 (70.7–75.1)	71.4 (66.8–76.0)	80.0 (72.3–87.7)	75.2 (73.8–76.7)	0.808 (0.778–0.837)	0.184 (0.173–0.195)
	Raw Interaction	73.6 (71.4–75.7)	71.6 (68.8–74.3)	80.9 (77.9–83.9)	75.9 (74.5–77.3)	0.813 (0.790–0.837)	0.183 (0.175–0.192)

Encoder Only indicates that predictions were generated using only the specified encoder. Without Encoder indicates that the specified encoder was excluded from the full model. Values are presented as the mean (95% confidence interval) across five folds. Accuracy, precision, recall, and F1-score are expressed as percentages.

**Table 16:** Classification performance of the fusion configurations under the two signal encoders.

Signal Encoder	Model	Accuracy	Precision	Recall	F1-Score	AUC	Brier Score	FPR at Sens. = 90%
2D ResNet-18	interaction early fusion	73.0 (69.3–76.7)	73.2 (69.4–76.9)	75.1 (68.5–81.7)	74.0 (70.1–78.0)	0.801 (0.750–0.852)	0.185 (0.160–0.209)	49.5 (41.1–57.9)
	hybrid fusion	74.8 (71.1–78.6)	76.4 (73.6–79.3)	73.9 (68.0–79.8)	75.1 (71.1–79.2)	0.820 (0.787–0.854)	0.176 (0.159–0.193)	50.5 (41.4–59.6)
EfficientNet-B0	interaction early fusion	73.7 (71.7–75.7)	72.1 (68.8–75.4)	80.2 (74.7–85.8)	75.8 (74.3–77.4)	0.815 (0.789–0.841)	0.179 (0.168–0.189)	47.1 (43.7–50.5)
	hybrid fusion	75.5 (73.3–77.7)	73.5 (69.3–77.7)	82.7 (74.3–91.1)	77.6 (75.1–80.1)	0.832 (0.809–0.856)	0.169 (0.157–0.181)	45.2 (37.4–53.1)

Values are reported as the mean (95% confidence interval) across five folds. Accuracy, precision, recall, and F1-score were computed at the Youden threshold determined on the validation set of each fold; FPR was computed at the threshold yielding 90% sensitivity. The EfficientNet-B0 rows correspond to the same models reported in Table 12. FPR, false positive rate; Sens., sensitivity.

**Table 17:** Computational complexity of the framework components under the two signal encoders.

Signal Encoder	Model	Parameters	Train (Min)	Memory (MB)	Batch 64 (ms)	Single (ms)
Shared	feature-only (MLP)	32,961	0.17	31	0.004	≈0.3
2D ResNet-18	signal-only	11,170,753	3.81	2075	0.31	≈1.4
	interaction early fusion	11,889,147	4.19	2288	0.37	≈3
	hybrid fusion	23,092,861	8.17	2288	0.68	≈4
EfficientNet-B0	signal-only	4,008,253	5.20	8247	0.59	≈5
	interaction early fusion	5,119,095	6.43	8449	0.65	≈6
	hybrid fusion	9,160,309	11.80	8449	1.24	≈11

Measured on one idle NVIDIA RTX A5000 (PyTorch 2.8.0) with inputs resident on the GPU, so data loading is excluded. Train is the epoch time multiplied by the mean number of epochs to early stopping, summed over the five folds. Memory is the peak during training at batch size 64 and includes the activations retained for backpropagation. Latency is the median of five repeated measurements, reported per record; single-record values are dominated by kernel launch overhead and varied by about 1.5 ms between repeats, so they are rounded. Hybrid fusion adds no parameters of its own: its parameters, training time and latency are the sums across the three branches, whereas its peak memory is the maximum.

**Table 18:** Distribution of the branch logits and their influence on the combined logit in the hybrid fusion model.

Branch	Logit Mean	Logit SD	AUC	Variance Share
signal-only	−0.017	1.838	0.798	0.466
feature-only	0.103	0.981	0.718	0.134
interaction early fusion	0.250	1.712	0.815	0.400

Logits were obtained from the test records of the five patient-level folds. AUC is the performance of each branch used on its own. The variance share is defined as $\mathrm{Cov}\left(z_{m},S\right)/\mathrm{Var}\left(S\right)$, where $z_{m}$ is the logit of branch *m* and *S* is the sum of the three branch logits; the shares sum to one across the branches.

**Table 19:** Comparison of combination strategies for the three branch logits.

Domain	Weighting	Accuracy	F1-Score	AUC	Brier Score
Logit	Equal (proposed)	75.5 (73.3–77.7)	77.6 (75.1–80.1)	0.832 (0.809–0.856)	0.169 (0.157–0.181)
	Tuned on validation	74.8 (72.8–76.9)	77.2 (75.5–78.9)	0.829 (0.803–0.855)	0.170 (0.158–0.182)
Probability	Equal	74.9 (72.3–77.6)	77.4 (75.0–79.8)	0.832 (0.807–0.856)	0.172 (0.161–0.183)
	Tuned on validation	74.6 (71.8–77.4)	77.7 (76.3–79.0)	0.828 (0.803–0.853)	0.172 (0.161–0.184)

Domain indicates whether the branch outputs were averaged as logits or as probabilities. Values are reported as the mean (95% confidence interval) across the five patient-level folds. Accuracy and F1-score are expressed as percentages. Strategies with tuned weighting were fitted using the validation records of each fold only.

**Table 20:** Performance of hybrid fusion under different thresholding strategies.

Threshold Type	Threshold	Accuracy	Precision	Recall	F1-Score	AUC	Brier Score	FPR
Default (0.5)	0.500 (0.500–0.500)	75.5 (74.4–76.6)	74.4 (71.7–77.1)	80.2 (72.2–88.1)	77.0 (74.6–79.4)	0.832 (0.809–0.856)	0.169 (0.157–0.181)	29.5 (22.6–36.3)
Youden	0.483 (0.383–0.583)	75.5 (73.3–77.7)	73.5 (69.3–77.7)	82.7 (74.3–91.1)	77.6 (75.1–80.1)	0.832 (0.809–0.856)	0.169 (0.157–0.181)	32.2 (22.2–42.2)
F1-Best	0.436 (0.338–0.534)	75.1 (72.1–78.2)	71.1 (66.8–75.3)	87.8 (84.3–91.2)	78.5 (77.0–79.9)	0.832 (0.809–0.856)	0.169 (0.157–0.181)	38.3 (28.5–48.1)
Sensitivity = 90%	0.391 (0.335–0.447)	73.2 (70.6–75.7)	68.1 (65.4–70.8)	90.5 (86.7–94.2)	77.6 (76.2–79.1)	0.832 (0.809–0.856)	0.169 (0.157–0.181)	45.2 (37.4–53.1)

Values are presented as the mean (95% confidence interval) across five folds. Accuracy, precision, recall, F1-score, and FPR are expressed as percentages. FPR, false positive rate.

**Table 21:** Statistical comparison between the signal-only and hybrid fusion models.

Metric	Pooled Difference (95% CI)	*p*-Value		
		Bootstrap	Paired *t*-Test	DeLong
Accuracy	0.026 (0.011–0.041)	0.002	0.068	—
Precision	0.004 (−0.011–0.020)	0.572	0.686	—
Recall	0.068 (0.047–0.089)	<0.001	0.253	—
F1-Score	0.034 (0.019–0.049)	<0.001	0.155	—
AUC	0.036 (0.027–0.045)	<0.001	0.003	<0.001
Brier Score	−0.017 (−0.022–−0.013)	<0.001	0.009	—

All metric differences, bootstrap confidence intervals, and bootstrap *p*-values were calculated from pooled out-of-fold predictions. Paired *t*-tests were performed using fold-level metric values, whereas the DeLong test was applied to the pooled AUC. All differences were calculated as hybrid fusion minus signal-only. A negative Brier score difference favors hybrid fusion because a lower Brier score indicates lower probabilistic prediction error. Differences are reported on the original metric scale, so a difference of 0.026 corresponds to 2.6 percentage points. CI, confidence interval.

**Table 22:** Subgroup performance of the final hybrid fusion model by sex and age.

Group	Subgroup	Mean *n*	Mean Stroke *n*	Accuracy	Precision	Recall	F1-Score	AUC	Brier Score
Sex	Female	183.4	87.2	74.5 (71.5–77.5)	70.6 (62.5–78.8)	81.4 (72.3–90.5)	75.1 (72.8–77.5)	0.826 (0.794–0.859)	0.172 (0.156–0.188)
	Male	233.2	127.2	76.3 (74.5–78.2)	75.6 (73.5–77.7)	83.7 (74.8–92.6)	79.3 (76.0–82.5)	0.837 (0.806–0.867)	0.166 (0.152–0.180)
Age	<65	182.8	72.4	76.4 (72.0–80.8)	68.9 (61.1–76.6)	74.5 (61.3–87.6)	71.1 (63.9–78.3)	0.834 (0.807–0.860)	0.165 (0.157–0.174)
	65–74	96.8	58.8	74.1 (66.6–81.6)	74.7 (67.1–82.3)	86.3 (76.3–96.3)	79.9 (72.7–87.1)	0.797 (0.725–0.869)	0.179 (0.158–0.200)
	≥75	137.0	83.2	75.0 (68.5–81.5)	75.9 (67.9–83.9)	86.9 (78.7–95.1)	80.8 (75.5–86.0)	0.812 (0.729–0.896)	0.167 (0.131–0.204)

Values are presented as the mean (95% confidence interval) across five folds. Accuracy, precision, recall, and F1-score are expressed as percentages. Mean *n* and Mean Stroke *n* denote the average subgroup and stroke record counts per fold, respectively.

**Table 23:** Dataset partitioning of the common-period cohort (2015–2019) for five-fold year-wise holdout evaluation.

Fold	Years		ECGs			Stroke in Test, *n* (%)
	Training	Held-Out	Training	Validation	Test	
1	2016–2019	2015	2712	210	210	75 (35.7)
2	2015, 2017–2019	2016	2641	245	246	100 (40.7)
3	2015–2016, 2018–2019	2017	2461	335	336	123 (36.6)
4	2015–2017, 2019	2018	2376	378	378	136 (36.0)
5	2015–2018	2019	2338	397	397	127 (32.0)

In each fold, ECGs from four calendar years were used for training, whereas ECGs from the held-out year were divided into validation and test sets.

**Table 24:** Classification performance on the common-period cohort under five-fold year-wise holdout evaluation.

Model	Accuracy	Precision	Recall	F1-Score	AUC	Brier Score
feature-only	62.3 (58.4–66.1)	49.5 (41.4–57.7)	59.6 (35.4–83.8)	52.0 (42.0–62.1)	0.694 (0.637–0.752)	0.226 (0.207–0.244)
signal-only	72.6 (67.2–78.0)	60.8 (49.1–72.5)	69.1 (49.8–88.4)	64.0 (52.5–75.4)	0.788 (0.745–0.830)	0.194 (0.174–0.213)
interaction early fusion	72.8 (68.8–76.8)	61.8 (53.1–70.5)	66.5 (52.8–80.3)	63.6 (56.2–70.9)	0.800 (0.775–0.825)	0.187 (0.166–0.209)
hybrid fusion	74.1 (72.5–75.7)	61.3 (56.8–65.8)	75.6 (67.1–84.1)	67.6 (61.9–73.4)	0.820 (0.786–0.854)	0.177 (0.159–0.194)

Values are presented as the mean (95% confidence interval) across the five year-wise holdout folds. Accuracy, precision, recall, and F1-score are expressed as percentages.

**Table 25:** Assessment of potential temporal confounding in stroke classification.

Model	Prediction Target	Evaluation Cohort	AUC
Year-Only	Stroke vs. non-stroke	2015–2019	0.490
Hybrid fusion	Stroke vs. non-stroke	Full period	0.832
Hybrid fusion	Stroke vs. non-stroke	2015–2019	0.821
Hybrid fusion	Common vs. non-common period	Stroke only	0.533

Values are reported as the mean AUC across five folds.

## Data Availability

Restrictions apply to the availability of these data. The data contain sensitive patient information and were obtained from Chuncheon Sacred Heart Hospital under an institutional data use agreement. The data are available from the corresponding author upon reasonable request and with the permission of Chuncheon Sacred Heart Hospital. The source code used for data preprocessing, model implementation, training, and evaluation is publicly available at https://github.com/ss990321/stroke-multimodal (accessed on 30 September 2026).

## Abbreviations

The following abbreviations are used in this manuscript:

AR	Autoregressive
AUC	Area Under the Receiver Operating Characteristic Curve
CI	Confidence Interval
CNN	Convolutional Neural Network
CT	Computed Tomography
DALYs	Disability-Adjusted Life Years
ECG	Electrocardiography

FN	False Negative
FP	False Positive
FPR	False Positive Rate
Grad-CAM	Gradient-weighted Class Activation Mapping
HRV	Heart Rate Variability
IRB	Institutional Review Board
KNN	*k*-Nearest Neighbors
MAR	Missing at Random
MBConv	Mobile Inverted Bottleneck Convolution
MCAR	Missing Completely at Random
MI	Missingness Indicator
MLP	Multilayer Perceptron
MNAR	Missing Not at Random
MRI	Magnetic Resonance Imaging
OR	Odds Ratio
ROC	Receiver Operating Characteristic
SHAP	SHapley Additive exPlanations
TN	True Negative
TP	True Positive
TPR	True Positive Rate
